# Ab Initio Vibro-Polaritonic Spectra in Strongly Coupled Cavity-Molecule Systems

**DOI:** 10.1021/acs.jctc.3c01135

**Published:** 2023-12-12

**Authors:** Thomas Schnappinger, Markus Kowalewski

**Affiliations:** Department of Physics, Stockholm University, AlbaNova University Center, SE-106 91 Stockholm, Sweden

## Abstract

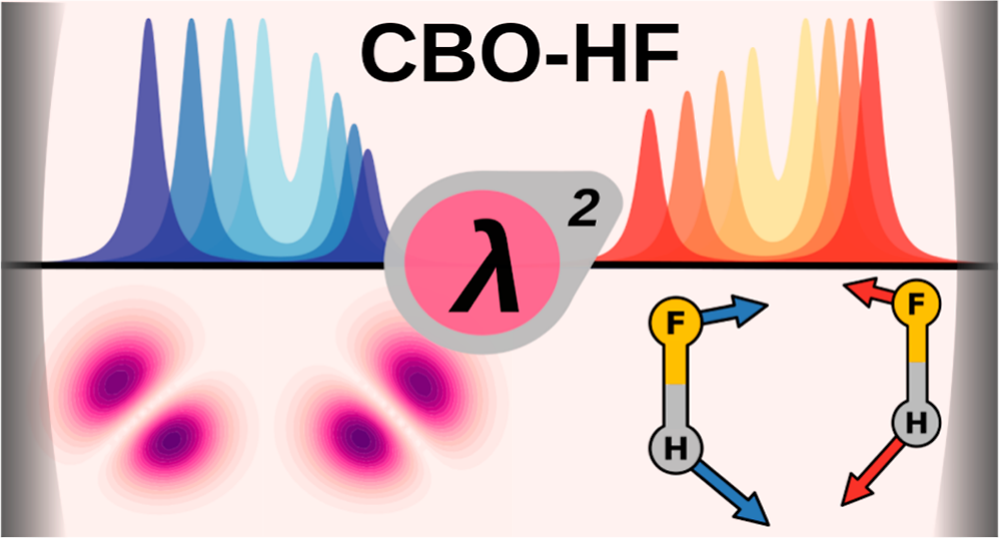

Recent experiments have revealed the profound effect of strong light–matter interactions in optical cavities on the electronic ground state of molecular systems. This phenomenon, known as vibrational strong coupling, can modify reaction rates and induce the formation of molecular vibrational polaritons, hybrid states involving both photon modes, and vibrational modes of molecules. We present an ab initio methodology based on the cavity Born–Oppenheimer Hartree–Fock ansatz, which is specifically powerful for ensembles of molecules, to calculate vibro-polaritonic IR spectra. This method allows for a comprehensive analysis of these hybrid states. Our semiclassical approach, validated against full quantum simulations, reproduces key features of the vibro-polaritonic spectra. The underlying analytic gradients also allow for optimization of cavity-coupled molecular systems and performing semiclassical dynamics simulations.

## 1 Introduction

Strong light–matter coupling between a molecular system and the electromagnetic field of an optical cavity offers new possibilities to modify chemical reactivity and selectivity, as demonstrated in recent experiments.^1−12^ A particularly intriguing but not yet fully understood situation occurs when a cavity mode is strongly coupled to molecular vibrations, called vibrational-strong coupling (VSC).^13−19^ For VSC the rate constants of ground state reactions can be modified even without external driving, i.e., without explicitly adding photons to the cavity. Probably one of the most striking features observed in such experiments is the change of the vibrational spectra due to the formation of molecular vibrational polaritons, hybrid states that involve both cavity modes and vibrational modes of molecules. These vibrational polaritons are characterized by the appearance of a lower polariton (LP) transition and a upper polariton (UP) transition separated by the Rabi splitting frequency, Ω_R_, originating from a single vibrational peak in the spectrum of the uncoupled molecular system. Both the presence of vibrational polaritons and the change in chemical reactivity may be characterized by a sharp resonance,^20−22^ which can be achieved if one of the cavity frequencies ω_c_ is in resonance with a vibrational mode in the reaction mixture.

Despite the large number of theoretical studies in the literature, the understanding of the underlying microscopic and macroscopic physical mechanisms, especially with respect to the effects of VSC, is still incomplete and under discussion.^15−19,23−25^ To better understand the formation of molecular vibrational polaritons and their role in polaritonic chemistry, a reliable theoretical description is needed that can also accurately treat the in practice most relevant case of collective strong coupling. One way to achieve such a description is to perform time-dependent simulations in the VSC regime that capture the dynamics of the system and yield optical spectra and changes in chemical reactivity.^26−29^ These explicit time domain calculations have their advantages in simulating complex and anharmonic dynamics but suffer from high computational cost, especially when the dynamics are calculated in an ab initio framework. Following the idea of Bonini and Flick,^30^ the use of the generalized force-constant matrix, often called the mass-weighted Hessian matrix ***H***^M^ in quantum chemistry, offers an alternative way to obtain vibro-polaritonic IR spectra without simulating the temporal evolution of the system. Within the well-known harmonic approximation, the eigenvectors and eigenvalues of ***H***^M^ give the vibro-polaritonic normal modes of the correlated matter-photon system and the frequencies of the vibro-polaritons.

In our recent work,^19^ we have introduced a Hartree–Fock ansatz in the cavity Born–Oppenheimer approximation (CBOA),^26,31−33^ capable of describing the electronic ground state of single molecules as well as of an ensemble of molecules coupled to an optical cavity. Within the framework of this cavity Born–Oppenheimer Hartree–Fock (CBO-HF) ansatz, we now derive analytic expressions for the first derivatives of the energy with respect to the nuclear and photonic degrees of freedom. These analytic gradients are then used to construct the mass-weighted Hessian matrix ***H***^M^ via finite differences and subsequently, ab initio vibro-polaritonic IR spectra within the harmonic approximation are calculated. After the introduction of the CBO-HF formalism, the first part of this work describes how the analytic expressions for the first derivatives of the energy are obtained and used to calculate vibro-polaritonic IR spectra in the CBO-HF framework. Next, the vibro-polaritonic IR spectra in the harmonic approximation are carefully compared against a full quantum mechanical approach for the rather anharmonic case of one and two diatomic hydrogen fluoride HF molecules. Even in this rather simple molecular example, we observe that the interaction between an optical cavity and the molecule(s) leads to a detuning and a change in the optical length of the cavity for both the harmonic approximation and the full quantum treatment, in agreement with our recent findings.^18^ Since both the self-consistent field (SCF) treatment and the description of the full dipole self-energy (DSE) contribution are crucial to capture relevant aspects for the description of strongly coupled molecules and their chemical properties,^17−19,34,35^ we analyze their direct influence on the vibro-polaritonic IR spectra of small ensembles of HF molecules. In the last part of the article, we discuss vibro-polaritonic IR spectra for ammonia (NH_3_). Here, the interaction with the confined light mode of an optical cavity influences the whole vibrational spectrum not only by creating vibrational polaritons but also by allowing symmetry breaking.

## 2 Theory

The nonrelativistic Pauli–Fierz Hamiltonian^22,35−38^ in the length gauge and the dipole approximation is used to describe the interaction of a confined light mode with atoms, molecules, and condensed matter systems. In all cases studied, we neglect the spatial structure of the cavity field, assuming that all molecules experience the same field. If the quantized cavity modes are coupled via their characteristic frequencies to vibrational degrees of freedom of molecules, the situation is described as VSC, for which CBOA^26,31−33^ is a well-suited theoretical approach. Within CBOA, the cavity modes are grouped with the nuclei in a generalized Born–Huang expansion,^35,39^ and then one can subsequently solve the quantum problem of the electrons and then of the nuclei and photons. Here, we first focus on the electronic degrees of freedom that have been shown to be strongly affected even for cavity modes that are tuned to the vibrational degrees of freedom.^18,19^ In the following, bold symbols denote vectors, and atomic units (ℏ = 4πε_0_ = *m*_e_ = 1) are used throughout the paper, unless otherwise noted. For a single-mode cavity, the electronic CBOA Hamiltonian takes the form
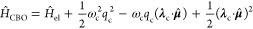
1where
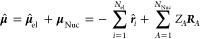
2represents the molecular dipole operator, which is defined by the operators of the *N*_el_ electron coordinates ***r*^**, the classic coordinates ***R*** of the *N*_Nuc_ nuclei, and the nuclear charges *Z*_A_.  is the Hamiltonian for the field-free many-electron system, and the second term defines the harmonic potential introduced by the photon displacement field, with the photon displacement coordinate *q*_c_ and ω_c_ being the frequency of the cavity mode. The third term of eq 1 describes the dipole coupling between the molecular system and the photon displacement field, which is characterized by coupling strength λ_c_. The last term is the DSE operator,^17,34,40^ which is an energy contribution that describes the self-polarization of the molecule-cavity system. Note that special attention must be paid to charged systems since the Hamiltonian in eq 1 becomes origin-dependent. In this situation, the quantities obtained are only meaningful with respect to a well-defined origin, for example, the center of mass. The coupling parameter λ_c_ for a cavity with an effective mode volume *V*_c_ is defined as follows
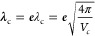
3

The unit vector ***e*** denotes the polarization axis of the cavity mode.

### 2.1 CBO-Hartree–Fock

In our recent work,^19^ we have introduced the CBO-HF approach which represents a formulation of the well-known Hartree–Fock ansatz in the context of CBOA. The resulting energy expectation value ⟨*E*_CBO_⟩ consists of four energy contributions
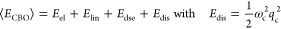
4

The first term *E*_el_ contains all Hartree–Fock energy components of the many-electron system^41,42^ and *E*_lin_ describes the linear dipole coupling between the photon displacement field, the electrons, and the nuclei. The energy contribution *E*_dse_ is due to the DSE operator in the Hamiltonian and *E*_dis_ is the energy resulting from the photon displacement field.^34^ Following the standard procedure,^41,42^ the transformation of ⟨*E*_CBO_⟩ in the basis of atomic orbitals results in
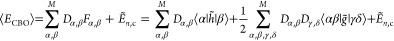
5where the indices α, β, γ, δ denote atomic orbitals, *F*_αβ_ the CBO-HF Fock matrix, and *D*_αβ_ the corresponding density matrix elements. Due to the classical nature of the nuclei and the photon displacement field in CBOA, when we determine the electronic ground state, all their energy contributions are scalar and summed up in 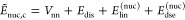
6

Modified one-electron integrals  and two-electron integrals  used to build the CBO-HF Fock matrix elements *F*_αβ_ consist of the standard one-electron integral ⟨α|*ĥ*|β⟩ and two-electron integrals ⟨αβ|*ĝ*|γδ⟩ as well as terms describing the linear cavity–electron interaction and the electronic DSE contributions
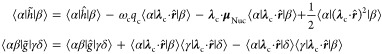
7

### 2.2 CBO-Hartree–Fock Gradients

The first derivative of the energy ⟨*E*_CBO_⟩ with respect to a nuclear or photon displacement coordinate ζ_*i*_ is the following.
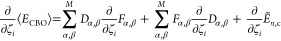
8

The first term in eq 8 is the Hellmann–Feynman term, the second term is the wave function derivative, or Pulay term, and the last part is the derivative of all scalar energy contributions. As the CBO-HF wave function is optimized based on the variational principle, the explicit calculation of the density matrix derivatives can be avoided^43^ and the Pulay term can be written in terms of orbital energies ϵ_*i*_, orbital coefficients *c*_*i*_, and overlap integral derivatives
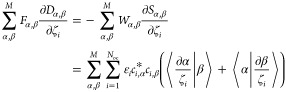
9

In the case of ζ_*i*_ being a photon displacement coordinate, eq 9 is zero, since the atomic orbitals α and β are independent of *q*_c_. A detailed derivation of all new terms, introduced by the field mode in eq 8 for ζ_I_ = *R*_*i*_ and ζ_I_ = *q*_c_, can be found in Section S1 of the Supporting Information. All derivatives for ⟨*ĥ*⟩, ⟨*ĝ*⟩, and *V*_nn_ with respect to nuclear coordinates are well-known and can be found elsewhere in the literature, for example in ref (42). The resulting analytic CBO-HF gradient ***g***_CBO_ is a (3*N*_A_ + 1) vector with *N*_A_ being the number of atoms in the molecule
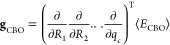
10

Note that we only consider a single cavity mode in the following. However, extending the technique is straightforward.

### 2.3 CBO-Hartree–Fock Frequencies

In order to calculate frequencies in the harmonic approximation for the CBO-HF ansatz, we need to calculate the Hessian matrix, ***H***, with dimensions (3*N*_A_ + 1) × (3*N*_A_ + 1) for the case of a single photon mode. Each element ***H***_*ij*_ is the second derivative of ⟨*E*_CBO_⟩ with respect to nuclear coordinates, respectively, the photon displacement coordinate^30^ that is
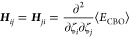
11

Using the analytic CBO-HF gradient ***g***_CBO_ the second derivatives can be determined using finite differences
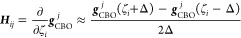
12

The harmonic frequencies and corresponding normal modes can be obtained by transforming Hessian matrix ***H*** from the ζ_*i*_ coordinates into mass-weighted version ***H***^M^ and subsequently solving the eigenvalue problem
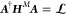
13

Here  is the diagonal matrix of eigenvalues _*i*_ and ***A*** is the matrix that diagonalizes the mass-weighted Hessian ***H***^M^. ***A*** is formed by juxtaposing its eigenvectors ***a***_*i*_ that define the normal modes. From the (3*N*_A_ + 1) eigenvalues, only (3*N*_A_ + 1 – 6), or (3*N*_A_ + 1 – 5) if the molecule is linear, corresponds to the harmonic frequencies of vibrational motions, whereas the others correspond to translations and rotations and are projected out. The harmonic frequencies, which are given in units of cm^–1^, are defined by
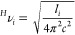
14

The corresponding intensities  in the harmonic approximation are calculated as the projection of the dipole moment gradient on the normal mode vectors ***a***_*i*_
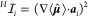
15

The necessary dipole moment gradient ∇⟨**μ̂**⟩ is calculated via finite differences. To validate the quality of the harmonic approximation and go beyond the semiclassical treatment, we determine the vibrational spectra of the cavity-coupled systems by then also determining the quantum mechanical wave function χ_*j*_ of the nuclei and photons in the Born–Huang expansion. Therefore, we have access not only to fundamental transitions but also to overtones and the anharmonicities of the potential. The anharmonic frequency ^A^ν_*j*_ for a given fundamental transition is the difference between the energies of the eigenstate *j* and the nuclear-photonic ground state. The corresponding intensity  is calculated with the nuclear-photonic eigenfunctions χ_*i*_ of the coupled molecular-cavity system
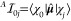
16

## 3 Computational Details

The analytic gradients for the CBO-HF ansatz have been implemented in the Psi4NumPy environment,^44^ which is an extension of the PSI4^45^ electronic structure package. All calculations were performed using the aug-cc-pVDZ basis set^46^ and the geometry of the isolated single HF and NH_3_ molecule have been optimized at the Hartree–Fock level of theory. Consistent with our previous studies,^18,19^ the optimized structure of a single HF molecule is used to define small ensembles of up to four molecules to study the collective effects on the infrared spectra of molecules in cavities. These ensembles are constructed by placing replicas of the single HF molecule separated by 800 Å inside the cavity. Two different orientations of the molecular HF ensembles are studied: The *all-parallel* configuration, in which all HF molecules are aligned parallel and the *antiparallel* configuration, which is characterized by a pairwise antiparallel orientation of the *N*_mol_ HF molecules. These constructed ensembles of *N*_mol_ HF molecules are placed at the maximum of the cavity field and oriented parallel with respect to the polarization axis of the cavity. For NH_3_ only the single molecule case is studied. As discussed in the Supporting Information of ref (19), the aligned orientation of HF is not the most energetically favorable when coupled to a cavity, but a transition state along the rotation with respect to the polarization axis. However, the two corresponding minima lead to a situation in which the molecule is not coupled to the cavity field. For the case of NH_3,_ the reoptimization in the presence of the cavity is characterized mainly by a rotation in the laboratory frame, resulting in an orientation with reduced coupling. Since the effects on the internal coordinates of HF and NH_3_ are small for the coupling strengths studied, we do not reoptimize the geometries of the molecular systems in the cavity and align the molecules to guarantee maximum coupling to the cavity. In all CBO-HF calculations performed in this work, we consider a single mode, lossless cavity. We keep the collective coupling strength λ_c_ constant by applying a scaling factor of  to obtain a fixed Rabi splitting for different ensemble sizes and treat λ_0_ as a tunable coupling parameter
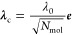
17

Here λ_0_ is equivalent to λ_c_ in eq 3 in the single molecule case. As a result, we increase the mode volume *V*_c_ of the cavity, but by including more molecules, we keep the average density of molecules *N*_mol_/*V*_c_ fixed. We use an artificially increased coupling strength λ_0_ in the range of 0.004 to 0.04, which corresponds to effective mode volumes, see eq 3, in the single-molecule case as large as 125.27 nm^3^ (for λ_0_ = 0.004) or as small as 1.25 nm^3^ (for λ_0_ = 0.04).

To validate the harmonic approximation, the vibrational spectra of a single HF molecule and a pair of HF molecules coupled to a cavity are calculated in a fully quantum mechanical way. By scanning along the bond length of each HF molecule and the photon displacement coordinate, we construct two-dimensional and three-dimensional CBO-HF cavity potential energy surface (cPES) and the corresponding dipole moment surfaces. These raw surfaces are interpolated to an equally spaced grid of 128 × 64 grid points, respectively, 128 × 128 × 64 grid points. A Gaussian shaped trial-function is numerically propagated in imaginary time^47^ (time step 0.1 au and 70,000 time steps) on the cPES with the Arnoldi propagation scheme^48^ to obtain the first four nuclear-photonic eigenfunctions χ_*i*_. The eigenenergies of the ground states and the first excited state are used to determine the fundamental transition energy, and the corresponding intensity is calculated according to eq 16. All quantum dynamics simulations are performed with our in-house quantum dynamics code (QDng).

For all vibrational spectra shown in this work, the underlying signals are broadened by a Lorentzian function with a width of 10 cm^–1^. All calculations were performed in a reproducible environment using the Nix package manager together with NixOS-QChem^49^ (commit f5dad404) and Nixpkgs (nixpkgs, 22.11, commit 594ef126).

## 4 Results and Discussion

### 4.1 Validation of the Harmonic Approximation

**Figure 1:**
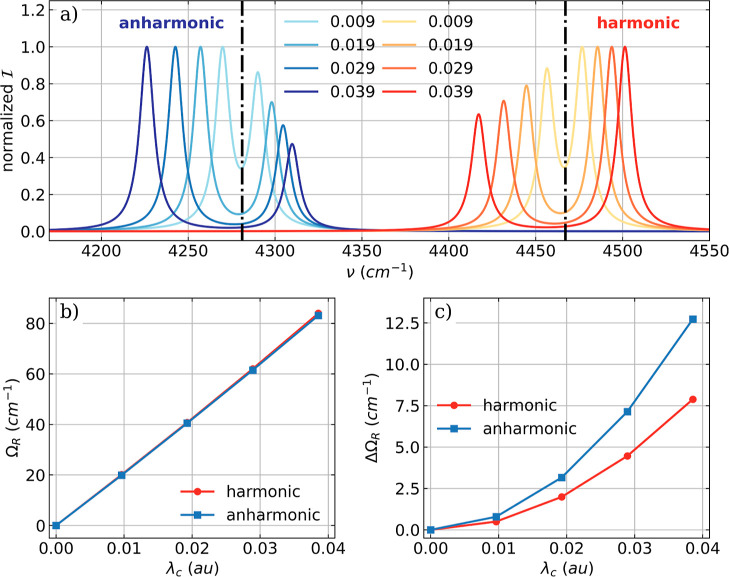
(a) Vibro-polaritonic IR spectra of a single HF molecule calculated in the harmonic approximation (reddish) and in the full-quantum setup (bluish), individually normalized for each coupling strength. The black dashed dotted lines indicate the bare molecular frequencies of the fundamental transitions of the harmonic case (^H^ν_1_ = 4467 cm^–1^) and the anharmonic case (^A^ν_1_ = 4281 cm^–1^). The cavity frequency ω_c_ is resonant with the corresponding fundamental transition, and the coupling strength λ_c_ increases from 0.009 to 0.039 au (from lightest to darkest color). The Rabi splitting Ω_R_ (b) and its asymmetry ΔΩ_R_ = ω_c_ – 0.5(ν^LP^ + ν^UP^) (c) as a function of λ_c_.

We begin with the validation of the harmonic approximation for vibro-polaritonic IR spectra of a single HF molecule coupled to an optical cavity. The spectra of the bare HF molecule calculated in the harmonic approximation and fully quantum mechanical (anharmonic) are shown in Figure S1 in the Supporting Information. The fundamental vibrational transition in the harmonic approximation has a frequency of ^H^ν_1_ = 4467 cm^–1^. The fully quantum mechanical treatment leads to an expected strong red shift of 186 cm^–1^ for the fundamental vibrational transition (^A^ν_1_ = 4281 cm^–1^). The vibro-polaritonic infrared spectra for different coupling strengths calculated in the harmonic approximation and full-quantum (anharmonic) as well as the trends in the Rabi splitting Ω_R_ are shown in Figure 1.

The resonant coupling of the cavity mode with the fundamental transition leads to the expected formation of a LP transition and UP transition in both the harmonic approximation and full-quantum treatment, see Figure 1a. The same anharmonic shift as in the field-free spectra is observed in the vibro-polaritonic spectra due to the anharmonicity along the nuclear coordinate independent of the coupling strength λ_c_ used. In addition to the red shift, the harmonic and full-anharmonic spectra differ mainly in their intensity patterns. In the harmonic approximation, the UP transition is more intense, and the difference between LP and UP is smaller compared to the full-quantum case. For the latter, the LP transition is more intense. We attribute this discrepancy to the fact that the intensities in the harmonic approximation are calculated only as the first derivative of the dipole moment. The increase in λ_c_ leads to a linear increase of the Rabi splitting Ω_R_ between LP and UP that is identical for both the harmonic and the full-quantum spectra (Figure 1b). Consistent with our previous work,^18^ we observe an asymmetry ΔΩ_R_ = ω_c_ – 0.5(ν^LP^ + ν^UP^) in the Rabi splitting (Figure 1c), where the LP is stronger red-shifted than the UP is blue-shifted with respect to ω_c_. This asymmetry is observed for the harmonic approximation and the full-quantum treatment, and in both cases, ΔΩ_R_ increases quadratically with λ_c_. However, the asymmetry is more pronounced in the full anharmonic case. This observed asymmetry of Ω_R_ can also be understood as detuning and change in the optical length of the cavity when interacting with the molecule. Similar results are found for two HF molecules in the *all-parallel* and *antiparallel* configurations coupled to an optical cavity. The corresponding vibro-polaritonic spectra for both cases are shown in Figures S2 and S3 of the Supporting Information. Due to the rescaling of λ_c_ (see eq 17) the spectra for the two molecule case look very similar to the single molecule case, and both configurations are nearly indistinguishable in their spectra.

**Figure 2:**
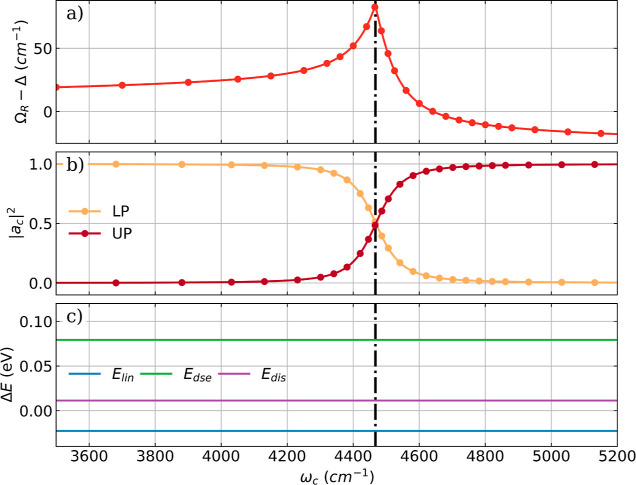
(a) Difference between the Rabi splitting Ω_R_ and Δ = ω_c_ – ^H^ν_1_ as a function of the cavity frequency ω_c_ for a single HF molecule using the CBO-HF harmonic approximation. Here Δ describes the difference between the cavity frequency ω_c_ and the fundamental bare molecular transition ^H^ν_1_. (b) The normal mode value |*a*_c_|^2^ describing the change in *q*_c_ for the LP and UP as a function of ω_c_. (c) Energy contributions due to cavity interaction as a function of the cavity frequency ω_c_ for the CBO-HF ansatz. Black dashed-dotted lines indicate the frequencies of the harmonic fundamental transition (^H^ν_1_ = 4467 cm^–1^). A constant coupling strength λ_c_ of 0.039 au is used.

To further extend the validation of the harmonic approximation, we next compared the effect of detuning the cavity frequency with respect to the fundamental transition of the single HF molecule on the vibro-polaritonic IR spectra. In addition to the spectral properties, the harmonic approximation gives access to normal modes ***a***_*i*_. In the standard Born–Oppenheimer approximation, these normal mode vectors describe the displacement of classical nuclei associated with the corresponding vibrational transition. In the CBOA for the case of the single photon mode, these vectors have an additional term *a*_c_ describing the change in the classical photon displacement field *q*_c_. The value of |*a*_c_|^2^ for a given normal mode is a measure of how strongly the corresponding vibrational transition interacts with the photon field. For an uncoupled light–matter system, a pure molecular transition is characterized by a |*a*_c_|^2^ value of zero, while the bare photon mode has a value of one. Note that due to the length gauge description *q*_c_ and the corresponding value *a*_c_ are no longer a pure photonic quantity if light and matter are coupled.^34,40,50^ Nevertheless, |*a*_c_|^2^ can still be used as a probe to identify how photonic the corresponding vibrational transition is. The information obtained is comparable to the coefficients in the Hopfield models.^51^ In Figure 2 the Rabi splitting Ω_R_ in harmonic approximation, the magnitude |*a*_c_|^2^ and the cavity-induced energy changes *E*_lin_, *E*_dse,_ and *E*_dis_ are plotted as a function of the cavity frequency ω_c_ for a fixed coupling strength.

**Figure 3:**
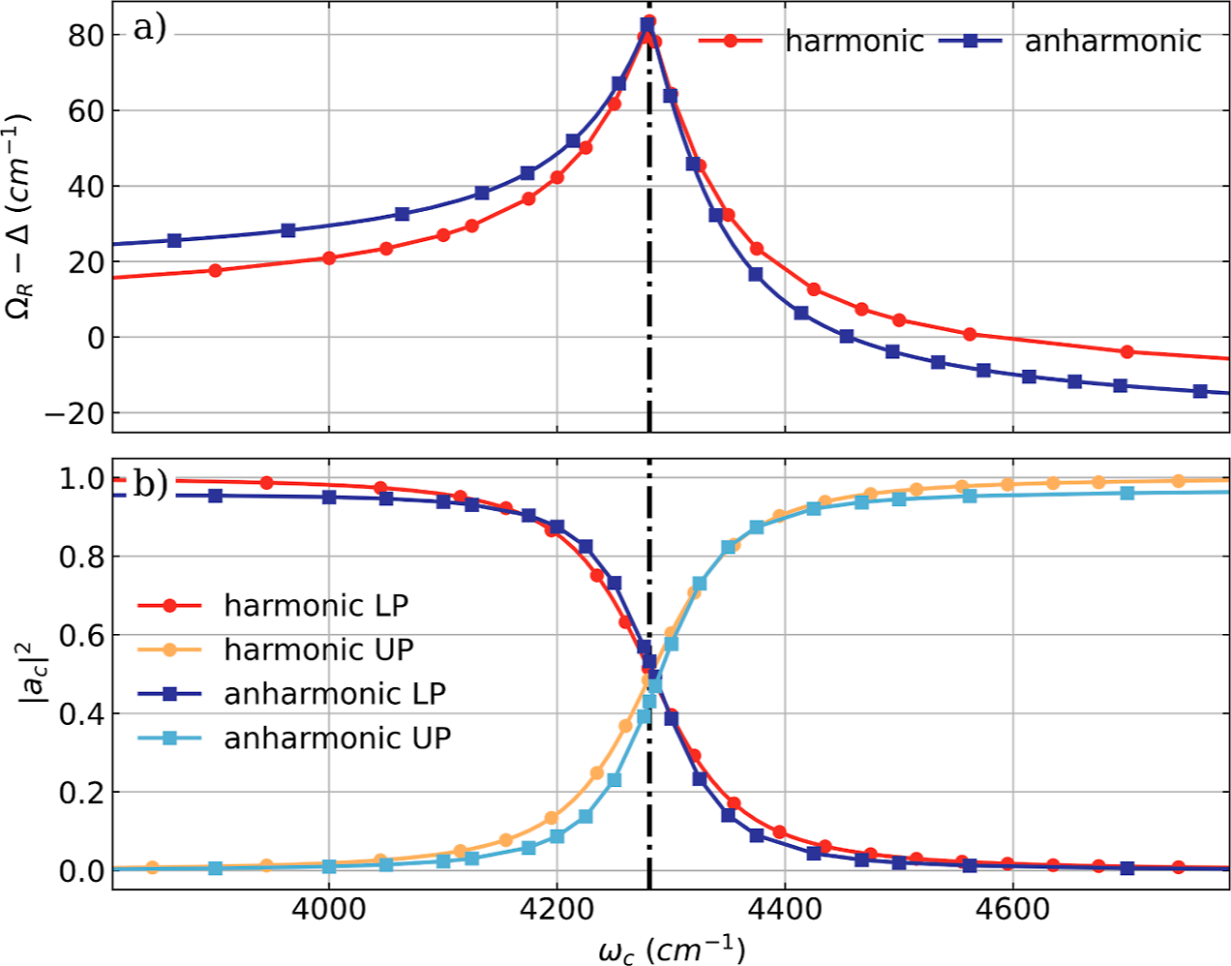
(a) Difference between the Rabi splitting Ω_R_ and Δ = ω_c_ – ^H^ν_1_ as a function of the cavity frequency ω_c_ for a single HF molecule in the full anharmonic treatment (blue) and the harmonic approximation (red). Here Δ describes the difference between the cavity frequency ω_c_ and the fundamental bare molecular transition ^H^ν_1_. (b) Change of the cavity normal mode magnitude |*a*_c_|^2^ (red) for LP and UP and the values  and  of the one-photon cavity vacuum state (blue). The harmonic results are shifted to match the anharmonic fundamental transition (^A^ν_1_ = 4281 cm^–1^), indicated by the black dashed dotted line. A constant coupling strength λ_c_ of 0.039 au is used.

For better visualization, the difference between Ω_R_ and Δ = ω_c_ – ^H^ν_1_, which describes the detuning of the cavity mode, is shown in Figure 3a. For an uncoupled molecular cavity system, this difference is zero and independent of ω_c_. Note that Ω_R_ – Δ is only a measure of the total size of Ω_R_, not of the asymmetry ΔΩ_R_, which is discussed in Figure 1. As expected, the largest Rabi splitting Ω_R_ is obtained for ω_c_ that is resonant with the fundamental bare molecular transition (^H^ν_1_). Interestingly, the difference between Ω_R_ and the detuning Δ is not symmetric with respect to ^H^ν_1_, and tends to a finite nonzero value even for large detunings. These results clearly show that the observation of a large Rabi splitting appears as a sharp resonance. A similar resonance is visible for the normal mode values |*a*_c_|^2^ of the vibrational transitions of the LP state and the UP state, as shown in Figure 3b. For ω_c_ equal to ^H^ν_1_ the |*a*_c_|^2^ values of LP and UP are close to 0.5, indicating that both vibrational transitions have a hybrid light–matter character. In contrast to the prominent resonance features observed in Figure 3a,b, the cavity-induced energy modifications *E*_lin_, *E*_dse_, and *E*_dis_ shown in Figure 3c are constant for all values of ω_c_. This result indicates that the energy changes induced by the cavity do not depend on any resonance condition, consistent with recent theoretical findings,^52,53^ where the coupling to a nonresonant cavity alters the ground state potential energy surface. Therefore, the experimentally observed change in chemical reactivity near resonance conditions cannot be explained by simple shifts of the underlying potential energy surfaces alone.

The direct comparison of the Rabi splitting and the light–matter character of the LP and UP transitions between the harmonic and full anharmonic simulations is shown in Figure 3. Since we treat the field displacement and the vibrational mode fully quantum mechanically, we have access to the nuclear-photonic eigenfunctions of the LP state (χ_LP_) and the UP state (χ_UP_). Examples of uncoupled and coupled nuclear-photonic eigenfunctions can be found in Section S3 of the Supporting Information in Figures S5 and S6. The coupled eigenfunctions can be expanded in terms of the uncoupled pure matter eigenfunction (χ_M_^(0)^) and the photonic eigenfunction (χ_C_^(0)^)
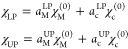
18

The absolute square of obtained expansion coefficients  and  can be used as a measure of the light–matter character similar to their classical pendant normal mode values |*a*_c_|^2^. As a side note, we want to clarify that this basis is sufficient to describe the hybridization between light and matter states, but it is not complete, since it does not take into account the contribution of the ground state wave function and of higher lying states. For a better comparison, the results of the harmonic simulations are red-shifted by 186.0 cm^–1^ to be aligned with the anharmonic simulation. The black dashed dotted line in Figure 3 indicates the anharmonic fundamental transition.

In good agreement with the previous discussion, the results obtained using the harmonic approximation are very similar to the results of the full-quantum simulations. After accounting for anharmonicity, the differences between Ω_R_ and detuning Δ (Figure 3a) are close to resonance and nearly identical and for large detunings qualitatively similar. The asymmetry ΔΩ_R_, as well as the asymmetry of the difference between Ω_R_ and Δ with respect to the fundamental transition, is more pronounced in the full-quantum simulation. Moreover, the |*a*_c_|^2^ values of LP and UP are, besides the overall anharmonic shift, in very good agreement between the harmonic approximation and the full anharmonic treatment. Overall, we can conclude that for the coupling strengths studied, the semiclassical harmonic approximation qualitatively reproduces the features of the vibro-polaritonic IR spectra well, even for a highly anharmonic molecule such as HF, except for the well-known general limitation of the harmonic approximation.^54,55^

Of course, the normal mode vectors ***a***_*i*_ obtained in the harmonic approximation contain more information than only the |*a*_c_|^2^ value discussed in this section. A detailed analysis of the whole ***a***_*i*_ vectors and a comparison with the nuclear-photonic eigenfunctions for the case of one HF molecule and two HF molecules can be found in Section S3 of the Supporting Information.

### 4.2 Influence of the SCF-Treatment and the Dipole Self-Energy on Vibro-Polaritonic Spectra

**Figure 4:**
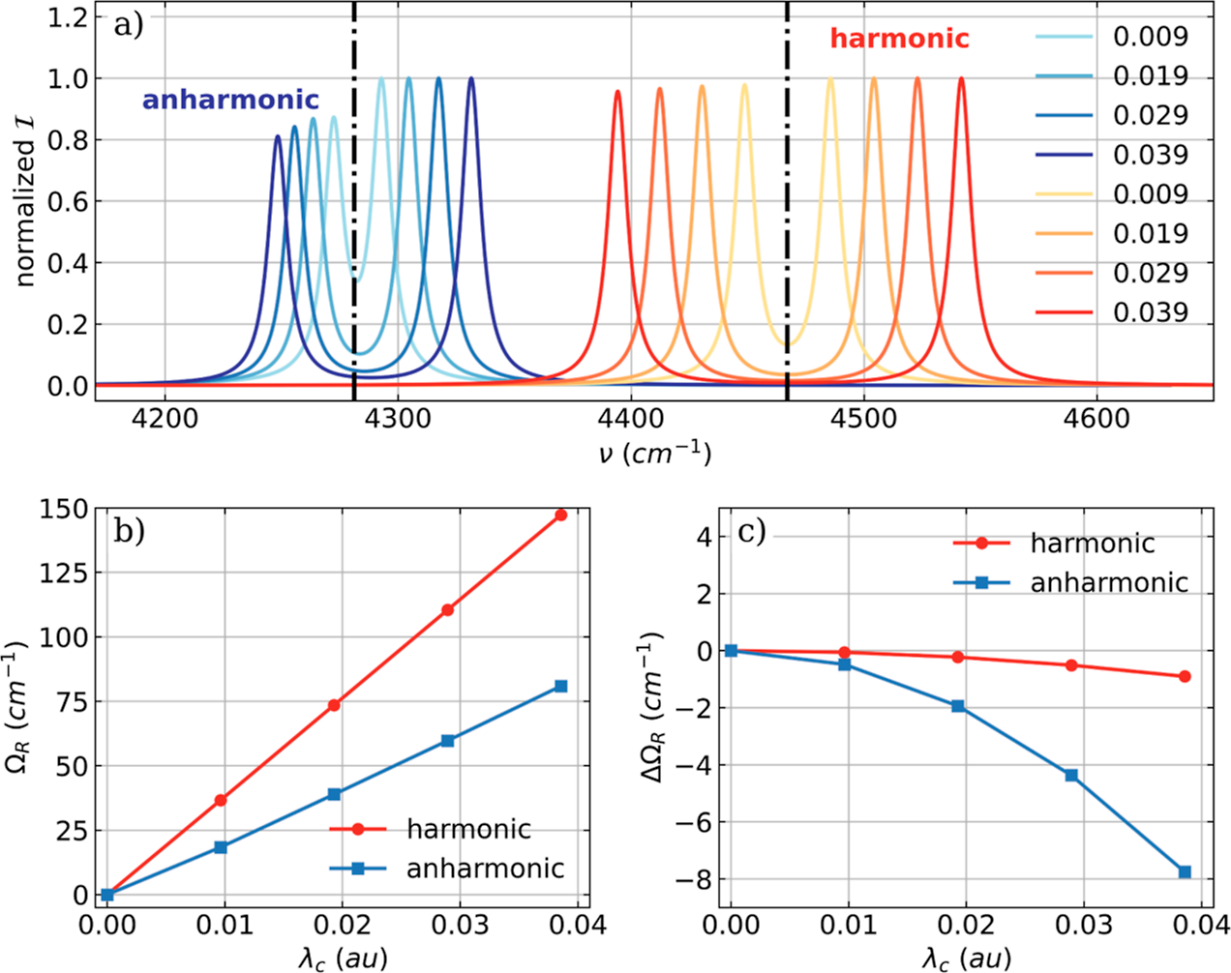
Vibro-polaritonic IR spectra of a single HF molecule calculated in the harmonic approximation (reddish) and in the full-quantum setup (bluish), individually normalized for each coupling strength. In both cases, the full SCF treatment is neglected; for details, see text. Black dashed-dotted lines indicate the frequencies of the harmonic fundamental transition (^H^ν_1_ = 4467 cm^–1^) and the anharmonic fundamental transition (^A^ν_1_ = 4281 cm^–1^). The cavity frequency ω_c_ is resonant with the corresponding fundamental transition in both cases, and the coupling strength λ_c_ increases from 0.009 to 0.039 au (from light to dark color). The Rabi splitting Ω_R_ (b) and its asymmetry ΔΩ_R_ = ω_c_ – 0.5(ν^LP^ + ν^UP^) (c) as a function of λ_c_.

As shown in our previous work,^18,19^ both self-consistent treatment and consideration of the full DSE operator are crucial to capture relevant aspects in the description of strongly coupled molecules and molecular ensembles. Therefore, we want to determine the influence of both factors on the vibro-polaritonic IR spectra for a single HF molecule and small ensembles with up to four HF molecules. To analyze the influence of the SCF treatment, the vibro-polaritonic IR spectra of a single HF molecule are calculated in the harmonic approximation and full quantum (anharmonic), neglecting the SCF treatment of the coupled electronic photonic system. For the harmonic approximation, this is achieved by using field-free dipole moments, density matrix elements *D*_αβ_, and orbital energies ϵ_*i*_ obtained by a standard Hartree–Fock calculation to determine the CBO-HF gradients and Hessians. In the full-anharmonic treatment, we use field-free Hartree–Fock energies and expectation values of dipole moments and DSE terms to construct the necessary potential energy surfaces in an extended molecular Jaynes–Cummings model (EJCM).^56−59^ The vibro-polaritonic infrared spectra obtained for different coupling strengths calculated in the harmonic approximation and the full anharmonic as well as the trends in Rabi splitting Ω_R_ are shown in Figure 4.

The comparison of the vibro-polaritonic IR spectra without SCF (Figure 4a) with those depicted in Figure 1a shows a qualitative difference for both the harmonic and the full anharmonic spectra. For the harmonic spectra, in particular, the SCF procedure yields a significantly different result. Without a fully converged CBO-HF the Rabi splitting Ω_R_ is larger, see Figure 4b, and nearly symmetric with respect to the cavity frequency, see Figure 4c. Furthermore, the intensities of the LP transition and the UP transition are nearly identical. For the full-anharmonic spectra in the EJCM model, the discrepancies are smaller but still visible. The value of Ω_R_ is reproduced quite well. However, its asymmetry ΔΩ_R_ is smaller and characterized by the blue-shift of LP and UP with respect to ω_c_. In addition, also the ratio of the intensities is inverted.

**Figure 5:**
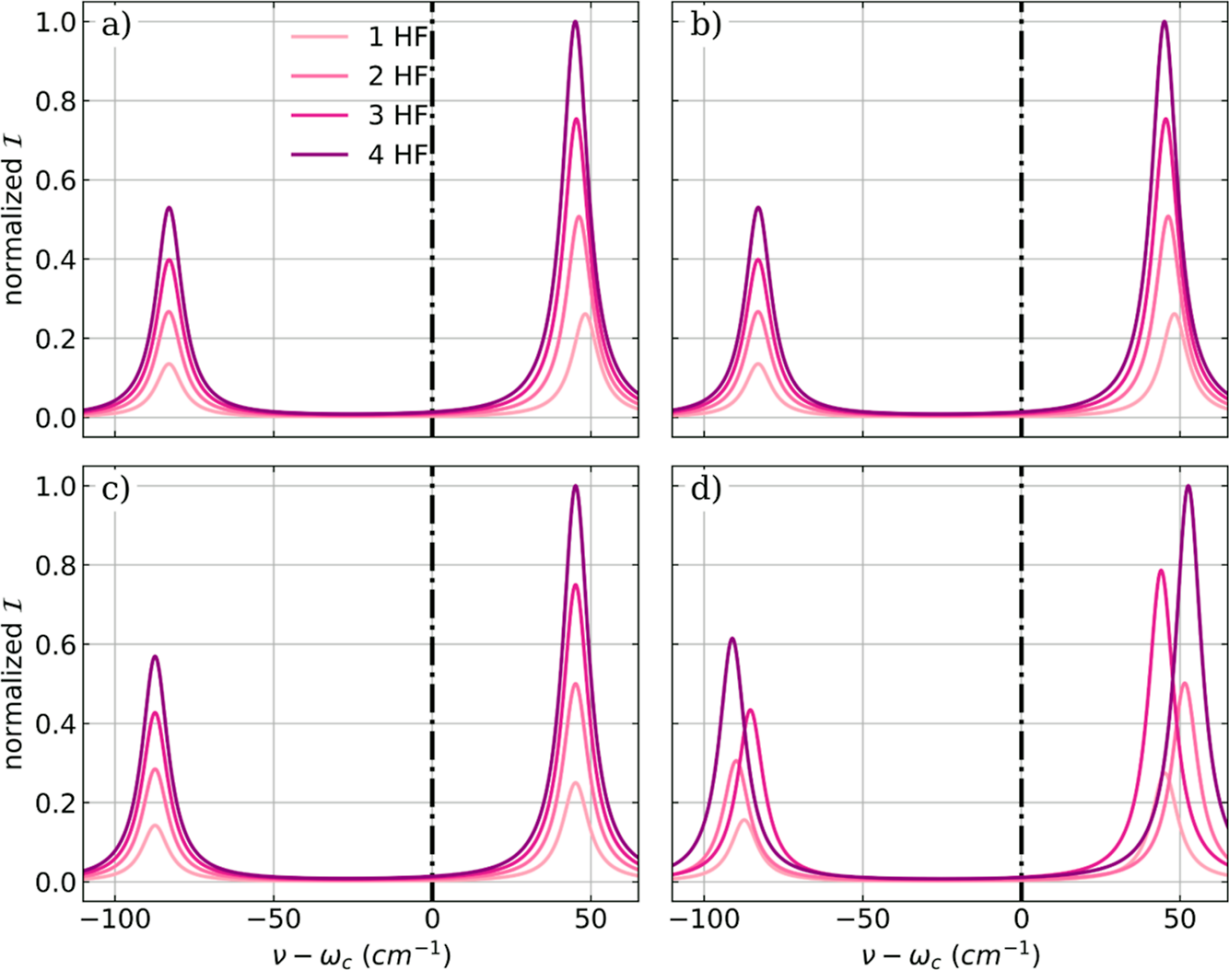
Vibro-polaritonic IR spectra calculated in the harmonic approximation for different numbers of HF molecules (color-coded) are shown with respect to the cavity frequency ω_c_. The cavity is resonant with the harmonic fundamental transition (^H^ν_1_ = 4467 cm^–1^, black dashed-dotted lines) and a rescaled coupling strength of λ_0_ of 0.057 au is used (see eq 17). (a) Full CBO-HF simulation in the *all-parallel* configuration. (b) Full CBO-HF simulation in the *antiparallel* configuration. (c) Linear CBO-HF simulation (without DSE terms) in the *all-parallel* configuration. (c) Linear CBO-HF simulation (without DSE terms) in the *antiparallel* configuration.

**Figure 6:**
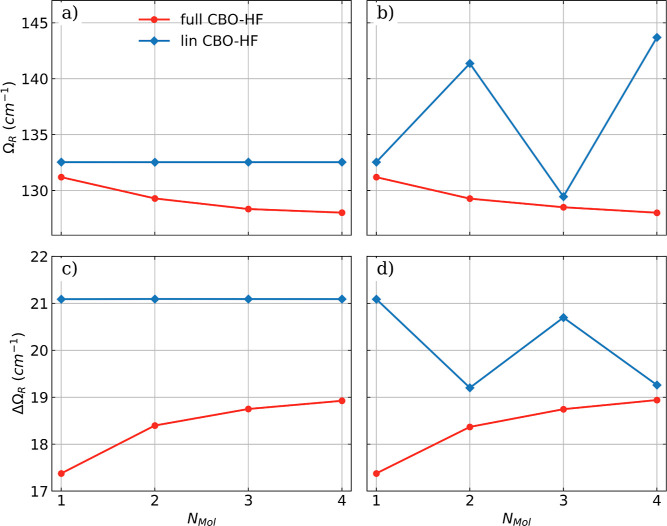
Rabi splitting Ω_R_ and asymmetry ΔΩ_R_ = ω_c_ – 0.5(ν^LP^ + ν^UP^) of the Rabi splitting as a function of *N*_Mol_. Both calculated in the harmonic approximation applying full CBO-HF and linear CBO-HF (without DSE terms) for the *all-parallel* configuration shown in (a,c) and in the *antiparallel* orientation shown in (b,d). The cavity frequency ω_c_ is resonant with the harmonic fundamental transition (4467 cm^–1^) and a rescaled coupling strength of λ_0_ of 0.057 au is used.

Another important aspect in describing VSC for molecules is the DSE, which gives rise to a cavity-induced interaction between molecules in an ensemble and is very sensitive to the molecular orientation.^18,19,60^ To quantify its influence on spectral features, we calculated the vibro-polaritonic IR spectra in the harmonic approximation for different numbers of HF molecules with and without the DSE terms included in the underlying CBO-HF ansatz. In the following, the case without the DSE terms is called linear CBO-HF. The results for the *all-parallel* and the *antiparallel* configuration are shown in Figure 5. The observed Rabi splitting Ω_R_ and its asymmetry ΔΩ_R_ for the *all-parallel* and the *antiparallel* configurations with and without the DSE term are shown as a function of the number of molecules in Figure 6.

The full CBO-HF spectra of the *all-parallel* configuration (Figure 5a) and the *antiparallel* configuration (Figure 5b) are indistinguishable. In line with our recent work^18^ increasing the number of molecules (*N*_Mol_) in the cavity while keeping the density constant (rescaling of the coupling strength by , see eq 17) leads to an increasing asymmetry ΔΩ_R_ in the splitting, see Figure 6c,d red lines. At the same time, the Rabi splitting itself decreases (see Figure 6a,b red lines), although the applied rescaling should keep it constant. This may be an effect of the rather large coupling strength applied. For weaker coupling, the applied rescaling of the coupling strength leads to an almost constant value of Ω_R_ as the number of molecules increases; see ref (18). If the DSE contribution is neglected, the two studied configurations become distinguishable; see Figure 5c,d. When the *all-parallel* spectra are compared with and without DSE, the differences are rather small. The observed Rabi splitting Ω_R_ is slightly larger in the case without DSE and independent of the number of molecules, as shown in Figure 6a blue line. Furthermore, the asymmetry ΔΩ_R_ is also independent of *N*_Mol_ and constant (Figure 6c blue line). The observed changes are more drastic in the case of the *antiparallel* configuration. In Figure 5d an alternating shift of the LP signal and the UP signal is observed when the number of molecules. Consequently, this alternation can be found in the Rabi splitting Ω_R_ and its asymmetry as an oscillation, see Figure 6b,d blue lines. The oscillating pattern can be attributed to the fact that the odd or even number of molecules in the *antiparallel* configuration creates two different situations. For even *N*_Mol_ the whole ensemble reduces to an effective *antiparallel* bimolecular case, and the situation of odd *N*_Mol_ is equivalent to that in the case of a single molecule. Consistent with the other works^17−19,34,40,61^ these results demonstrate the importance of the DSE terms and the SCF treatment for obtaining a physically consistent result.

### 4.3 Beyond Diatomic Molecules: Vibro-Polaritonic Spectra of NH_3_

**Figure 7:**
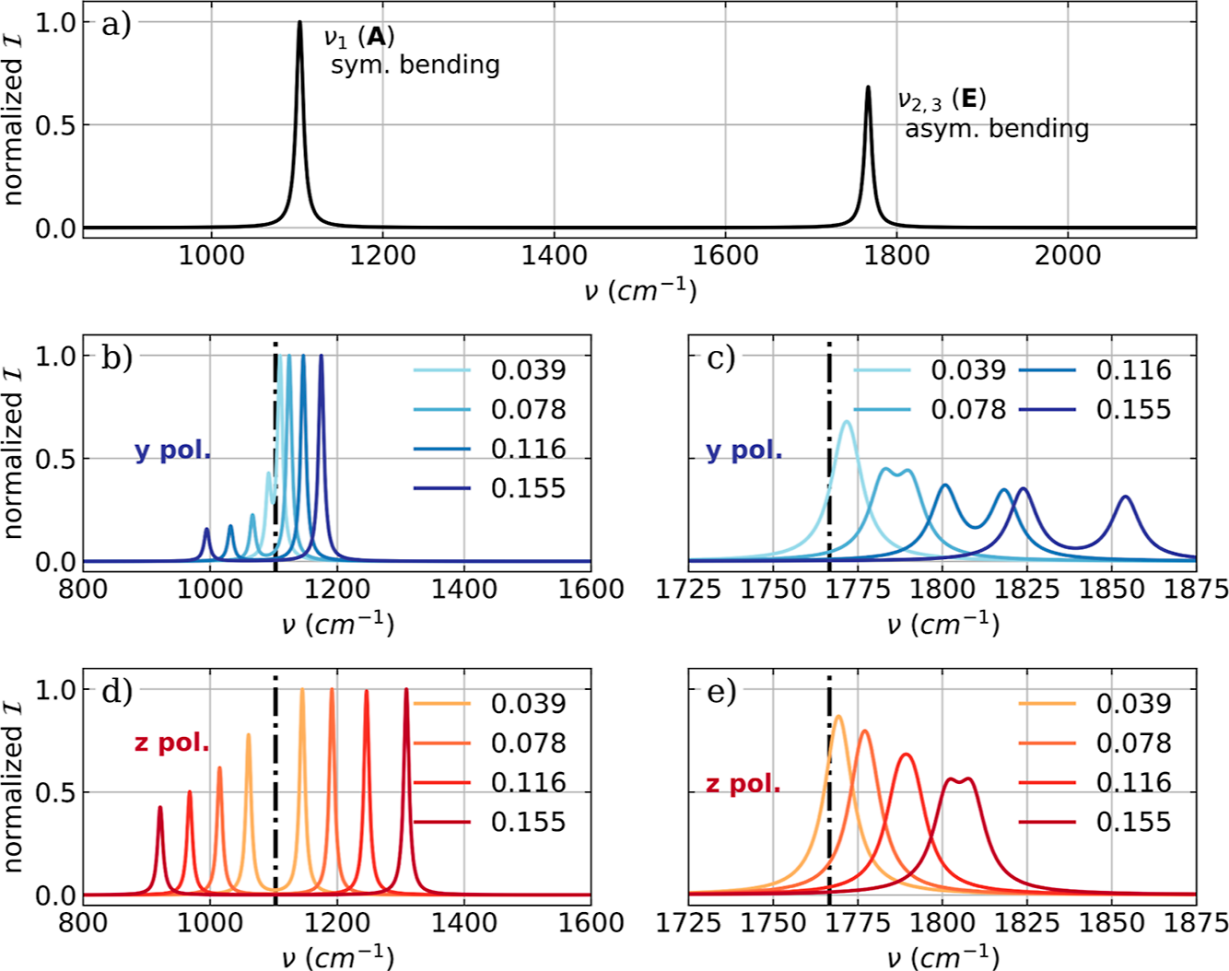
(a) Vibronic IR spectra of a single NH_3_ molecule calculated in the harmonic approximation. The low energy part of the vibro-polaritonic IR spectra of a single NH_3_ molecule zoomed into the symmetric mode (b,d) and the two asymmetric bending modes shown in (c,e). The polarization axis of the cavity mode is the *y* axis for (b,c) and equal to the *z* axis for (d,e). The cavity frequency ω_c_ is resonant with the symmetric bending mode (1103 cm^–1^) and the cavity field strength λ_c_ increases from 0.039 to 0.155 au.

So far, we have discussed only diatomic molecules, which have only one vibrational degree of freedom or, in the case of the ensembles, linear combinations of the same vibrational degree. However, since we have validated the harmonic approximation and the associated normal-mode analysis, its semiclassical nature also allows us to treat larger molecular systems. Therefore, in the last part of this work, we will study the vibro-polaritonic IR spectra of a single NH_3_ molecule. The bare NH_3_ molecule has six vibrational degrees of freedom: One symmetric bending mode (ν_1_), two degenerate asymmetric bending modes (ν_2,3_), one symmetric stretching mode (ν_4_), and two degenerate asymmetric stretching modes (ν_5,6_). Since NH_3_ is not a linear molecule and the vibrational modes have different spatial orientations (symmetry), the polarization axis of the single cavity mode plays an important role. In our simulations, the single NH_3_ molecule is oriented with respect to the center of nuclear charges and the *C*_3_ symmetry axis is aligned with the *z* axis of the laboratory frame. Since for this chosen molecular orientation the *x* axis and the *y* axis are equivalent, only the *y* polarization and the *z* polarization of the single cavity mode are discussed in the following. The cavity frequency ω_c_ is set to be resonant with the symmetric bending mode ν_1_, which has the lowest energy of 1103 cm^–1^ and is the strongest transition in the field-free case. The bare molecular vibronic IR spectrum and the vibro-polaritonic IR spectra of a single NH_3_ molecule are shown in Figure 7 for the bending modes and in Figure S14 of the Supporting Information for the stretching modes.

As expected, the splitting into a LP and a UP transition is observed for the symmetric bending mode ν_1_ when coupled to a resonant optical cavity (see Figure 7b,d). The Rabi splitting Ω_R_ increases with increasing coupling strength and is larger for *z* polarization than for *x*/*y* polarization due to the chosen molecular orientation. Although not in resonance with the selected cavity frequency, the signals of the asymmetric bending modes (ν_2_ and ν_3_) are also affected by the coupling to the cavity mode, as shown in Figure 7c,e. For the *x*/*y* polarization the signal is blue-shifted and for a coupling strength λ_c_ larger than 0.078 au the degeneracy of the two transitions is lifted. This shift and the splitting into two separate signals are also observed for the *z* polarization. However, it is smaller for this polarization and occurs only at higher coupling strengths.

**Figure 8:**
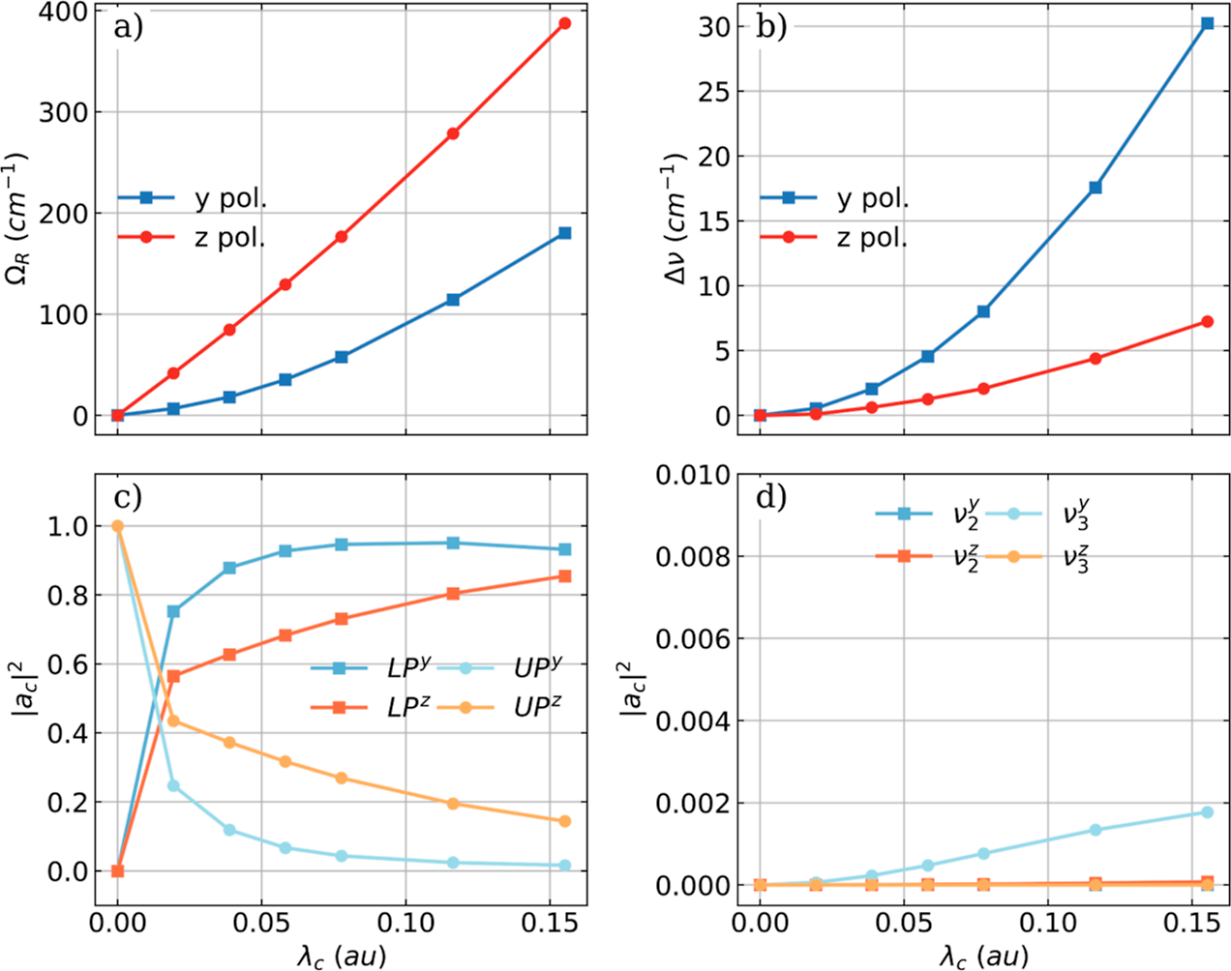
(a) Rabi splitting Ω_R_ for the symmetric bending mode of a single NH_3_ molecule as a function of λ_c_. (b) Splitting Δν of the asymmetric bending modes due to cavity interaction. The normal mode value |*a*_c_|^2^ describing the change in *q*_c_ for the UP and LP modes (c) and for the two asymmetric bending modes (d). The cavity frequency ω_c_ is resonant with the symmetric bending mode (1103 cm^–1^) and both the *y*/*x* (bluish) and the *z* (reddish) polarizations of the cavity mode are shown.

To better understand and characterize the two observed splittings in the vibro-polaritonic IR spectra of NH_3_, their magnitude is plotted for both polarization directions as a function of the coupling strength λ_c_ in Figure 8a,b. Furthermore, the corresponding values |*a*_c_|^2^ for the associated normal modes for both splittings are plotted in Figure 8c,d as a function of λ_c_. These |*a*_c_|^2^ values describe the change in the classical photon displacement field *q*_c_ and probe how photonic the corresponding vibrational transition is.

Clearly, the formation of a LP and a UP transition for the symmetric bending mode is more efficient for the *z* polarization since the observed Rabi splitting Ω_R_ is larger and scales almost linear with ϵ_c_, see Figure 8a. For the *y*/*x* polarization the value is smaller, but the scaling is close to quadratic. The stronger effect observed for *z* polarization is understandable, since the symmetric bending mode mainly involves motion along the *z* axis in the laboratory frame and only very small changes along the *y*/*x* direction. A closer look at the |*a*_c_|^2^ values for the LP and UP normal modes (Figure 8c) shows behavior similar to that of the case of the HF molecule. Once the cavity coupling is present, the |*a*_*c*_|^2^ values are clearly nonzero for both LP and UP. Since for the *z* polarization the coupling between the cavity mode and the symmetric bending mode is more efficient, the |*a*_c_|^2^ value of the UP mode is closer to that of the LP mode and clearly larger compared to the *y*/*x* case. In other words, the light and molecule are less hybridized for the *y*/*x* polarization. The splitting and the |*a*_c_|^2^ values for the two asymmetric bending modes shown in Figure 8b,d behave differently. In the *z* polarization, where a smaller splitting is observed, both |*a*_c_|^2^ values are lower than 0.001. Even for the *y*/*x* case, they do not become larger than 0.002, which is still orders of magnitude smaller than for the LP and the UP transition. This is clear evidence that both asymmetric bending modes mostly are matter transitions, and no light–matter hybrid states are formed. The observed splitting of ν_2_ and ν_3_ when coupled to a cavity can be explained by a symmetry breaking by the confined photon mode. Its polarization axis in *x* or *y* makes these two directions, which are identical in the case without a cavity, distinguishable. Note that in our current simulations we only consider a single photon mode, and in a more general setup, there are many modes with usually more than one polarization direction. Therefore, we assume that the observed splitting due to symmetry breaking is overestimated in our calculations. However, the fact that interaction with an optical cavity not only affects near-resonance transitions by forming LP and UP states, but also modifies all signals by slightly changing the energetics and breaking molecular symmetries is probably still important in more general cases and was already observed earlier in literature.^30^

## 5 Summary and Conclusions

Based on the recently formulated cavity Born–Oppenheimer Hartree–Fock ansatz^19^ we have introduced a wave function-based methodology to calculate the vibro-polaritonic IR spectra in an ab initio manner. By applying the well-known harmonic approximation, we have access to the vibrational frequencies and normal modes of systems when light and matter are strongly coupled. Using the cavity Born–Oppenheimer approximation, the obtained normal modes combine both nuclear and photonic degrees of freedom, allowing a detailed analysis of the vibro-polariton states. The necessary second derivatives of the CBO-HF energy are calculated via finite differences of the analytic first derivatives (gradients) introduced in this work.

We demonstrate the capability of our framework by carefully comparing the vibro-polaritonic IR spectra obtained for small ensembles of HF molecules with full quantum-mechanical calculations. Overall, the semiclassical harmonic approximation qualitatively reproduces the main features of the vibro-polaritonic IR spectra very well. It even captures to some extent polarization effects such as cavity detuning previously observed^18^ when the full DSE contribution is included and the coupled electronic-photonic problem is solved self-consistently. The semiclassical nature of the harmonic approximation allows efficient description of vibro-polaritonic IR spectra of large molecular systems or small ensembles. As a first test case, we simulated the vibro-polaritonic spectra of a single NH_3_ molecule. In addition to the expected formation of vibro-polaritonic states, the coupling to an optical cavity changes the whole vibrational spectrum. The presence of the confined light mode leads to an energy shift of most signals, and depending on the cavity polarization and the molecular orientation, the molecular symmetry is reduced.

The new analytical gradients used here to obtain vibro-polaritonic IR spectra open many avenues for future exploration. The gradients and numerical Hessian can be used to optimize the molecular system coupled to an optical cavity. In contrast to the cavity/field-free case, rotation with respect to the laboratory frame is no longer trivial for optimizations in the presence of a cavity mode with a defined polarization axis.^19^ Beside the possibility to optimize molecules under VSC, the analytical gradients can be used to perform ab initio semiclassical dynamics simulations for single molecules or small ensembles.

## Supporting Information Available

Derivation of the CBO-HF energy gradients, discussion of HF and NH_3_ coupled to an optical cavity, and a detailed comparison of nuclear-photonic eigenfunctions and vibro-polaritonic normal modes (PDF)
All data underlying this study are available from the corresponding author upon reasonable request

## References

[ref1] Hutchison J. A.; Schwartz T.; Genet C.; Devaux E.; Ebbesen T. W. Modifying chemical landscapes by coupling to vacuum fields. Angew. Chem., Int. Ed. Engl. 2012, 51, 1592–1596. 10.1002/anie.201107033. 22234987

[ref2] George J.; Chervy T.; Shalabney A.; Devaux E.; Hiura H.; Genet C.; Ebbesen T. W. Multiple Rabi Splittings under Ultrastrong Vibrational Coupling. Phys. Rev. Lett. 2016, 117, 153601 10.1103/PhysRevLett.117.153601. 27768350

[ref3] Thomas A.; George J.; Shalabney A.; Dryzhakov M.; Varma S. J.; Moran J.; Chervy T.; Zhong X.; Devaux E.; Genet C.; Hutchison J. A.; Ebbesen T. W. Ground-State Chemical Reactivity under Vibrational Coupling to the Vacuum Electromagnetic Field. Angew. Chem., Int. Ed. Engl. 2016, 55, 11462–11466. 10.1002/anie.201605504. 27529831 PMC5113700

[ref4] Thomas A.; Lethuillier-Karl L.; Nagarajan K.; Vergauwe R. M. A.; George J.; Chervy T.; Shalabney A.; Devaux E.; Genet C.; Moran J.; Ebbesen T. W. Tilting a ground-state reactivity landscape by vibrational strong coupling. Science 2019, 363, 615–619. 10.1126/science.aau7742. 30733414

[ref5] Hirai K.; Hutchison J. A.; Uji-I H. Recent Progress in Vibropolaritonic Chemistry. ChemPlusChem 2020, 85, 1981–1988. 10.1002/cplu.202000411. 32869494

[ref6] Hirai K.; Takeda R.; Hutchison J. A.; Uji-I H. Modulation of Prins Cyclization by Vibrational Strong Coupling. Angew. Chem., Int. Ed. Engl. 2020, 59, 5332–5335. 10.1002/anie.201915632. 31970847

[ref7] Kumar S.; Biswas S.; Rashid U.; Mony K. S.; Vergauwe R. M. A.; Kaliginedi V.; Thomas A. Extraordinary Electrical Conductance of Non-conducting Polymers Under Vibrational Strong Coupling. arXiv 2023, arXiv:2303.03777 preprint 10.48550/arXiv.2303.03777. 38736166

[ref8] Ahn W.; Triana J. F.; Recabal F.; Herrera F.; Simpkins B. S. Modification of ground-state chemical reactivity via light-matter coherence in infrared cavities. Science 2023, 380, 1165–1168. 10.1126/science.ade7147. 37319215

[ref9] Bhuyan R.; Mony J.; Kotov O.; Castellanos G. W.; Gómez Rivas J.; Shegai T. O.; Börjesson K. The Rise and Current Status of Polaritonic Photochemistry and Photophysics. Chem. Rev. 2023, 123, 10877–10919. 10.1021/acs.chemrev.2c00895. 37683254 PMC10540218

[ref10] Zhong C.; Hou S.; Zhao X.; Bai J.; Wang Z.; Gao F.; Guo J.; Zhang F. Driving DNA Origami Coassembling by Vibrational Strong Coupling in the Dark. ACS Photonics 2023, 10, 1618–1623. 10.1021/acsphotonics.3c00235.

[ref11] Gu K.; Si Q.; Li N.; Gao F.; Wang L.; Zhang F. Regulation of Recombinase Polymerase Amplification by Vibrational Strong Coupling of Water. ACS Photonics 2023, 10, 1633–1637. 10.1021/acsphotonics.3c00243.

[ref12] Weight B. M.; Krauss T. D.; Huo P. Investigating Molecular Exciton Polaritons Using Ab Initio Cavity Quantum Electrodynamics. J. Phys. Chem. Lett. 2023, 14, 5901–5913. 10.1021/acs.jpclett.3c01294. 37343178 PMC10316409

[ref13] Nagarajan K.; Thomas A.; Ebbesen T. W. Chemistry under Vibrational Strong Coupling. J. Am. Chem. Soc. 2021, 143, 16877–16889. 10.1021/jacs.1c07420. 34609858

[ref14] Dunkelberger A. D.; Simpkins B. S.; Vurgaftman I.; Owrutsky J. C. Vibration-Cavity Polariton Chemistry and Dynamics. Annu. Rev. Phys. Chem. 2022, 73, 429–451. 10.1146/annurev-physchem-082620-014627. 35081324

[ref15] Simpkins B. S.; Dunkelberger A. D.; Vurgaftman I. C. Control, Modulation, and Analytical Descriptions of Vibrational Strong Coupling. Chem. Rev. 2023, 123, 5020–5048. 10.1021/acs.chemrev.2c00774. 37018158

[ref16] Campos-Gonzalez-Angulo J. A.; Poh Y. R.; Du M.; Yuen-Zhou J. Swinging between Shine and Shadow: Theoretical Advances on Thermally-Activated Vibropolaritonic Chemistry (A Perspective). J. Chem. Phys. 2023, 158, 230901 10.1063/5.0143253. 37318163

[ref17] Sidler D.; Ruggenthaler M.; Schäfer C.; Ronca E.; Rubio A. A perspective on ab initio modeling of polaritonic chemistry: The role of non-equilibrium effects and quantum collectivity. J. Chem. Phys. 2022, 156, 230901 10.1063/5.0094956. 35732522

[ref18] Sidler D.; Schnappinger T.; Obzhirov A.; Ruggenthaler M.; Kowalewski M.; Rubio A. Unraveling a Cavity Induced Molecular Polarization Mechanism from Collective Vibrational Strong Coupling. arXiv 2023, arXiv:2306.06004 preprint 10.48550/arXiv.2306.06004. PMC11103705 38717382

[ref19] Schnappinger T.; Sidler D.; Ruggenthaler M.; Rubio A.; Kowalewski M. Cavity Born-Oppenheimer Hartree-Fock Ansatz: Light-Matter Properties of Strongly Coupled Molecular Ensembles. J. Phys. Chem. Lett. 2023, 14, 8024–8033. 10.1021/acs.jpclett.3c01842. 37651603 PMC10510432

[ref20] Li X.; Mandal A.; Huo P. Cavity frequency-dependent theory for vibrational polariton chemistry. Nat. Commun. 2021, 12, 1315 10.1038/s41467-021-21610-9. 33637720 PMC7910560

[ref21] Schäfer C.; Flick J.; Ronca E.; Narang P.; Rubio A. Shining light on the microscopic resonant mechanism responsible for cavity-mediated chemical reactivity. Nat. Commun. 2022, 13, 7817 10.1038/s41467-022-35363-6. 36535939 PMC9763331

[ref22] Lindoy L. P.; Mandal A.; Reichman D. R. Quantum dynamical effects of vibrational strong coupling in chemical reactivity. Nat. Commun. 2023, 14, 2733 10.1038/s41467-023-38368-x. 37173299 PMC10182063

[ref23] Schütz S.; Schachenmayer J.; Hagenmüller D.; Brennen G. K.; Volz T.; Sandoghdar V.; Ebbesen T. W.; Genes C.; Pupillo G. Ensemble-Induced Strong Light-Matter Coupling of a Single Quantum Emitter. Phys. Rev. Lett. 2020, 124, 113602 10.1103/PhysRevLett.124.113602. 32242709

[ref24] Davidsson E.; Kowalewski M. The Role of Dephasing for Dark State Coupling in a Molecular Tavis-Cummings Model. J. Chem. Phys. 2023, 159, 044306 10.1063/5.0155302. 37493131 PMC7615654

[ref25] Davidsson E.; Kowalewski M. Atom Assisted Photochemistry in Optical Cavities. J. Phys. Chem. A 2020, 124, 4672–4677. 10.1021/acs.jpca.0c03867. 32392061 PMC7294536

[ref26] Flick J.; Narang P. Cavity-Correlated Electron-Nuclear Dynamics from First Principles. Phys. Rev. Lett. 2018, 121, 113002 10.1103/PhysRevLett.121.113002. 30265119

[ref27] Schäfer C.; Flick J.; Ronca E.; Narang P.; Rubio A. Shining light on the microscopic resonant mechanism responsible for cavity-mediated chemical reactivity. Nat. Commun. 2022, 13, 7817 10.1038/s41467-022-35363-6. 36535939 PMC9763331

[ref28] Yu Q.; Hammes-Schiffer S. Multidimensional Quantum Dynamical Simulation of Infrared Spectra under Polaritonic Vibrational Strong Coupling. J. Phys. Chem. Lett. 2022, 13 (48), 11253–11261. 10.1021/acs.jpclett.2c03245. 36448842

[ref29] Mondal M. E.; Koessler E. R.; Provazza J.; Vamivakas A. N.; Cundiff S. T.; Krauss T. D.; Huo P. Quantum dynamics simulations of the 2D spectroscopy for exciton polaritons. J. Chem. Phys. 2023, 159, 159 10.1063/5.0166188. 37655761

[ref30] Bonini J.; Flick J. Ab Initio Linear-Response Approach to Vibro-Polaritons in the Cavity Born-Oppenheimer Approximation. J. Chem. Theory Comput. 2022, 18, 2764–2773. 10.1021/acs.jctc.1c01035. 35404591 PMC9097282

[ref31] Flick J.; Ruggenthaler M.; Appel H.; Rubio A. Atoms and molecules in cavities, from weak to strong coupling in quantum-electrodynamics (QED) chemistry. Proc. Natl. Acad. Sci. U.S.A. 2017, 114, 3026–3034. 10.1073/pnas.1615509114. 28275094 PMC5373338

[ref32] Flick J.; Appel H.; Ruggenthaler M.; Rubio A. Cavity Born–Oppenheimer approximation for correlated electron–nuclear-photon systems. J. Chem. Theory Comput. 2017, 13, 1616–1625. 10.1021/acs.jctc.6b01126. 28277664 PMC5390309

[ref33] Fischer E. W.; Saalfrank P. Beyond Cavity Born–Oppenheimer: On Nonadiabatic Coupling and Effective Ground State Hamiltonians in Vibro-Polaritonic Chemistry. J. Chem. Theory Comput. 2023, 19 (20), 7215–7229. 10.1021/acs.jctc.3c00708. 37793029

[ref34] Schäfer C.; Ruggenthaler M.; Rokaj V.; Rubio A. Relevance of the Quadratic Diamagnetic and Self-Polarization Terms in Cavity Quantum Electrodynamics. ACS Photonics 2020, 7, 975–990. 10.1021/acsphotonics.9b01649. 32322607 PMC7164385

[ref35] Ruggenthaler M.; Sidler D.; Rubio A. Understanding Polaritonic Chemistry from Ab Initio Quantum Electrodynamics. Chem. Rev. 2023, 123, 11191–11229. 10.1021/acs.chemrev.2c00788. 37729114 PMC10571044

[ref36] Spohn H. Dynamics of Charged Particles and Their Radiation Field; Cambridge University Press, 2004.

[ref37] Ruggenthaler M.; Tancogne-Dejean N.; Flick J.; Appel H.; Rubio A. From a quantum-electrodynamical light–matter description to novel spectroscopies. Nat. Rev. Chem 2018, 2, 0118–216. 10.1038/s41570-018-0118.

[ref38] Jestädt R.; Ruggenthaler M.; Oliveira M. J.; Rubio A.; Appel H. Light-matter interactions within the Ehrenfest–Maxwell–Pauli–Kohn–Sham framework: fundamentals, implementation, and nano-optical applications. Adv. Phys. 2019, 68, 225–333. 10.1080/00018732.2019.1695875.

[ref39] Schäfer C.; Ruggenthaler M.; Rubio A. Ab initio nonrelativistic quantum electrodynamics: Bridging quantum chemistry and quantum optics from weak to strong coupling. Phys. Rev. A 2018, 98, 043801 10.1103/PhysRevA.98.043801.

[ref40] Rokaj V.; Welakuh D. M.; Ruggenthaler M.; Rubio A. Light–matter interaction in the long-wavelength limit: no ground-state without dipole self-energy. J. Phys. B: At., Mol. Opt. Phys. 2018, 51, 034005 10.1088/1361-6455/aa9c99.

[ref41] Szabo A.; Ostlund N. S. Modern Quantum Chemistry: Introduction to Advanced Electronic Structure Theory, 1st ed.; Dover Publications, Inc.: Mineola, 1996.

[ref42] Jensen F. Introduction to Computational Chemistry, 3rd ed.; John Wiley & Sons: Nashville, TN, 2017.

[ref43] Pulay P. Ab initio calculation of force constants and equilibrium geometries in polyatomic molecules. Mol. Phys. 1969, 17, 197–204. 10.1080/00268976900100941.

[ref44] Smith D. G. A.; Burns L. A.; Sirianni D. A.; Nascimento D. R.; Kumar A.; James A. M.; Schriber J. B.; Zhang T.; Zhang B.; Abbott A. S.; Berquist E. J.; Lechner M. H.; Cunha L. A.; Heide A. G.; Waldrop J. M.; Takeshita T. Y.; Alenaizan A.; Neuhauser D.; King R. A.; Simmonett A. C.; Turney J. M.; Schaefer H. F.; Evangelista F. A.; DePrince A. E.; Crawford T. D.; Patkowski K.; Sherrill C. D. Psi4NumPy: An Interactive Quantum Chemistry Programming Environment for Reference Implementations and Rapid Development. J. Chem. Theory Comput. 2018, 14, 3504–3511. 10.1021/acs.jctc.8b00286. 29771539

[ref45] Smith D. G. A.; Burns L. A.; Simmonett A. C.; Parrish R. M.; Schieber M. C.; Galvelis R.; Kraus P.; Kruse H.; Di Remigio R.; Alenaizan A.; James A. M.; Lehtola S.; Misiewicz J. P.; Scheurer M.; Shaw R. A.; Schriber J. B.; Xie Y.; Glick Z. L.; Sirianni D. A.; O’Brien J. S.; Waldrop J. M.; Kumar A.; Hohenstein E. G.; Pritchard B. P.; Brooks B. R.; Schaefer H. F. 3rd; Sokolov A. Y.; Patkowski K.; DePrince A. E. 3rd; Bozkaya U.; King R. A.; Evangelista F. A.; Turney J. M.; Crawford T. D.; Sherrill C. D. Psi4 1.4: Open-source software for high-throughput quantum chemistry. J. Chem. Phys. 2020, 152, 184108 10.1063/5.0006002. 32414239 PMC7228781

[ref46] Kendall R. A.; Dunning T. H.; Harrison R. J. Electron affinities of the first?row atoms revisited. Systematic basis sets and wave functions. J. Chem. Phys. 1992, 96, 6796–6806. 10.1063/1.462569.

[ref47] Kosloff R.; Tal-Ezer H. A direct relaxation method for calculating eigenfunctions and eigenvalues of the schrödinger equation on a grid. Chem. Phys. Lett. 1986, 127, 223–230. 10.1016/0009-2614(86)80262-7.

[ref48] Smyth E. S.; Parker J. S.; Taylor K. T. Numerical integration of the time-dependent Schrödinger equation for laser-driven helium. Comput. Phys. Commun. 1998, 114, 1–14. 10.1016/S0010-4655(98)00083-6.

[ref49] Kowalewski M.; Seeber P. Sustainable packaging of quantum chemistry software with the Nix package manager. Int. J. Quantum Chem. 2022, 122, e26872 10.1002/qua.26872.

[ref50] Welakuh D. M.; Rokaj V.; Ruggenthaler M.; Rubio A. Non-perturbative Mass Renormalization Effects in Non-relativistic Quantum Electrodynamics. arXiv 2023, arXiv:2310.03213 preprint 10.48550/arXiv.2310.03213.

[ref51] Hopfield J. J. Theory of the Contribution of Excitons to the Complex Dielectric Constant of Crystals. Phys. Rev. 1958, 112, 1555–1567. 10.1103/PhysRev.112.1555.

[ref52] Pavosevic F.; Smith R. L.; Rubio A. Cavity Click Chemistry: Cavity-Catalyzed Azide-Alkyne Cycloaddition. J. Phys. Chem. A 2023, 10.1021/acs.jpca.3c06285. 37992280

[ref53] Pavošević F.; Smith R. L.; Rubio A. Computational study on the catalytic control of endo/exo Diels-Alder reactions by cavity quantum vacuum fluctuations. Nat. Commun. 2023, 14, 2766 10.1038/s41467-023-38474-w. 37179341 PMC10183045

[ref54] Bloino J.; Biczysko M. Reference Module in Chemistry, Molecular Sciences and Chemical Engineering; Elsevier, 2015.

[ref55] Raj A.; Chao Y.-B.; Witek H. A. Testing the limitations of harmonic approximation in the determination of Raman intensities. Mol. Phys. 2022, 120, e2069613 10.1080/00268976.2022.2069613.

[ref56] Jaynes E. T.; Cummings F. W. Comparison of quantum and semiclassical radiation theories with application to the beam maser. Proc. IEEE 1963, 51, 89–109. 10.1109/PROC.1963.1664.

[ref57] Kowalewski M.; Bennett K.; Mukamel S. Cavity Femtochemistry: Manipulating Nonadiabatic Dynamics at Avoided Crossings. J. Phys. Chem. Lett. 2016, 7, 2050–2054. 10.1021/acs.jpclett.6b00864. 27186666

[ref58] Gudem M.; Kowalewski M. Controlling the Photostability of Pyrrole with Optical Nanocavities. J. Phys. Chem. A 2021, 125, 1142–1151. 10.1021/acs.jpca.0c09252. 33464084 PMC7883346

[ref59] Couto R. C.; Kowalewski M. Suppressing non-radiative decay of photochromic organic molecular systems in the strong coupling regime. Phys. Chem. Chem. Phys. 2022, 24, 19199–19208. 10.1039/d2cp00774f. 35861014 PMC9382694

[ref60] Haugland T. S.; Philbin J. P.; Ghosh T. K.; Chen M.; Koch H.; Narang P. Understanding the Polaritonic Ground State in Cavity Quantum Electrodynamics. arXiv 2023, arXiv:2307.14822 preprint 10.48550/arXiv.2307.14822. 40377188

[ref61] Schnappinger T.; Kowalewski M. Nonadiabatic Wave Packet Dynamics with Ab Initio Cavity-Born-Oppenheimer Potential Energy Surfaces. J. Chem. Theory Comput. 2023, 19, 460–471. 10.1021/acs.jctc.2c01154. 36625723 PMC9878721

