# Correction to Ab Initio Vibro-Polaritonic Spectra in Strongly Coupled Cavity-Molecule Systems

**DOI:** 10.1021/acs.jctc.4c01374

**Published:** 2024-10-31

**Authors:** Thomas Schnappinger, Markus Kowalewski

Recently, we have discovered several errors in our paper “Ab Initio Vibro-Polaritonic Spectra in Strongly Coupled Cavity-Molecule Systems” [Schnappinger and Kowalewski J. Chem. Theory Comput.2023, 19, 927838084914 10.1021/acs.jctc.3c01135PMC10753771] which we would like to correct here. The expression of the infrared (IR) intensity  for the harmonic approximation given in eq 15 is lacking the atomic masses and should read instead
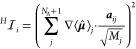
1with the dipole moment gradient  and the normal mode vectors ***a***_*i*_ rescaled by the square root of the corresponding atomic mass *M*_*j*_. In addition, the IR intensity  in the anharmonic case needs to be squared
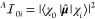
2We have recalculated every spectrum shown in the manuscript and Supporting Information using the correct version of the intensities. In this correction, we provide a new version of all affected figures (see Figure 1, Figure 4, Figure 5, Figure 7, and Figures S3, S4, and S13). This only changes the intensities of the lower polariton (LP) and upper polariton (UP) signals in the case of the harmonic approximation. This change in the harmonic intensities leads to a better agreement between the IR spectra calculated in the harmonic approximation and in the full quantum setup. We explicitly point out that these corrections do not change the main results and conclusions of the original manuscript.

**Figure 1:**
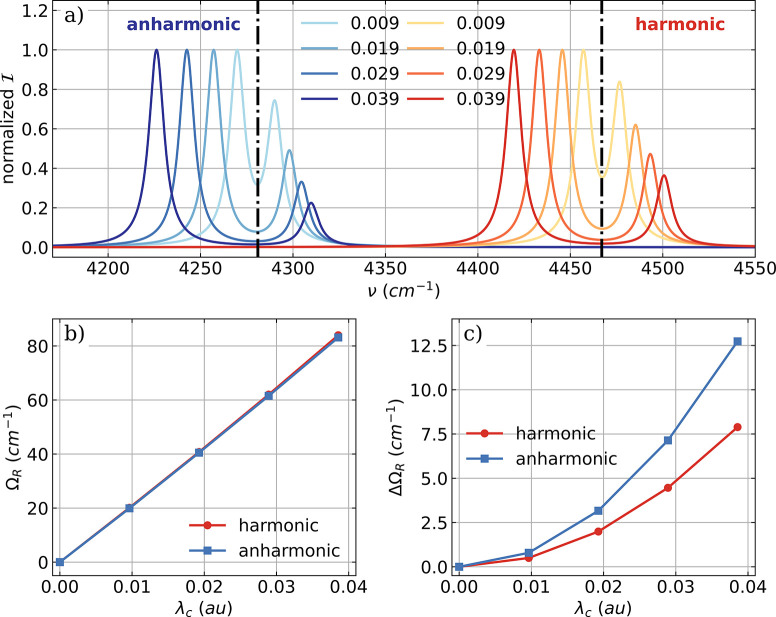
a) Vibro-polaritonic IR spectra of a single HF molecule calculated in the harmonic approximation (reddish) and in the full-quantum setup (bluish), individually normalized for each coupling strength. The black dashed dotted lines indicate the bare molecular frequencies of the fundamental transitions of the harmonic case (^*H*^ν_1_ = 4467 cm^–1^) and the anharmonic case (^*A*^ν_1_ = 4281 cm^–1^). The cavity frequency ω_*c*_ is resonant with the corresponding fundamental transition, and the coupling strength λ_*c*_ increases from 0.009 au to 0.039 au (from lightest to darkest color). The Rabi splitting Ω_*R*_ (b) and its asymmetry ΔΩ_*R*_ =ω_*c*_ – 0.5(ν^*LP*^+ν^*UP*^) (c) as a function of λ_*c*_.

**Figure 4:**
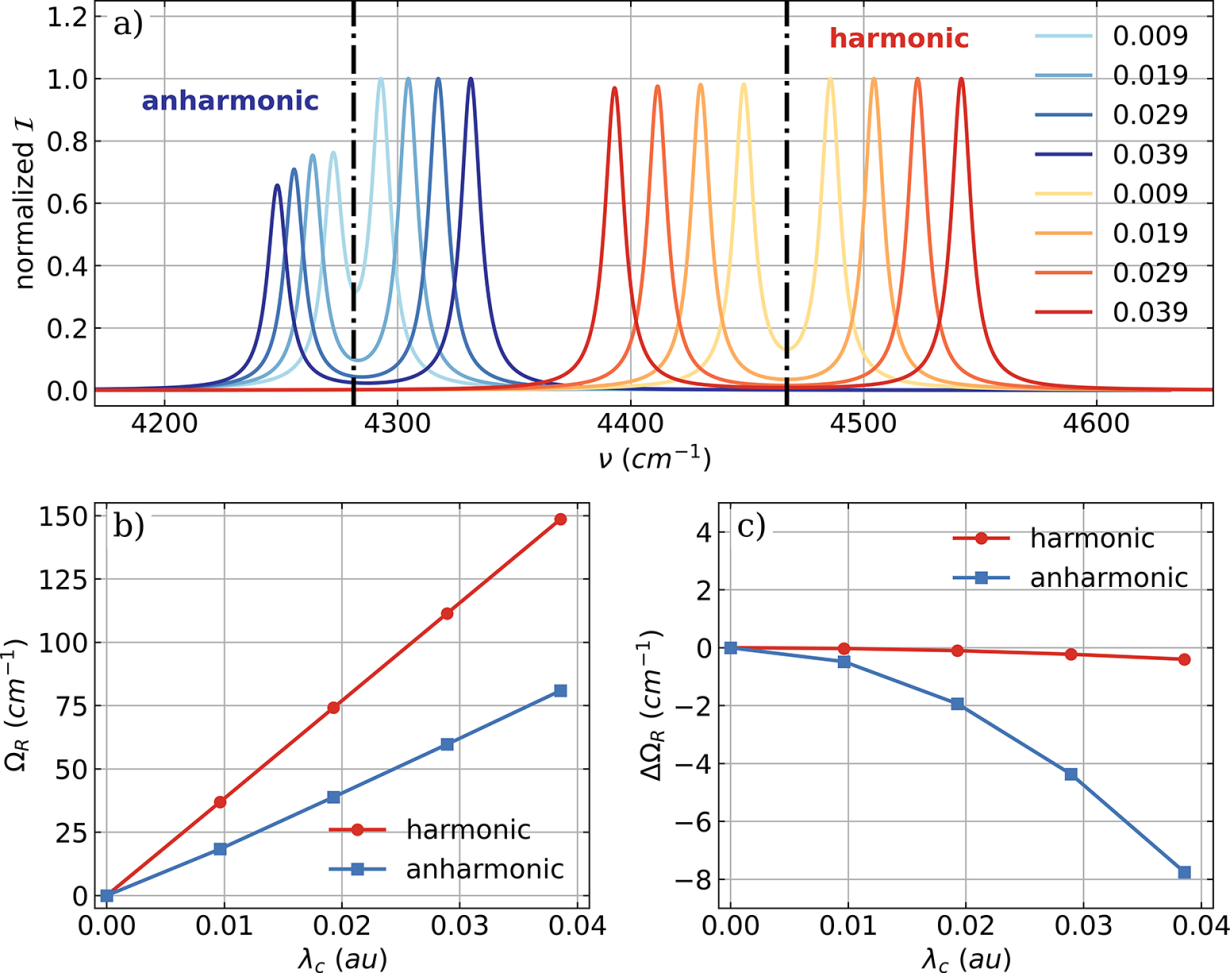
a) Vibro-polaritonic IR spectra of a single HF molecule calculated in the harmonic approximation (reddish) and in the full-quantum setup (bluish), individually normalized for each coupling strength. In both cases, the full SCF treatment is neglected; for details, see text. Black dashed-dotted lines indicate the frequencies of the harmonic fundamental transition (^*H*^ν_1_ = 4467 cm^–1^) and the anharmonic fundamental transition (^*A*^ν_1_ = 4281 cm^–1^). The cavity frequency ω_*c*_ is resonant with the corresponding fundamental transition in both cases, and the coupling strength λ_*c*_ increases from 0.009 au to 0.039 au (from light to dark color). b) Rabi splitting Ω_*R*_ as a function of λ_*c*_. c) Asymmetry ΔΩ_*R*_ = ω_*c*_ – 0.5(ν^*LP*^+ν^*UP*^) of the Rabi splitting.

**Figure 5:**
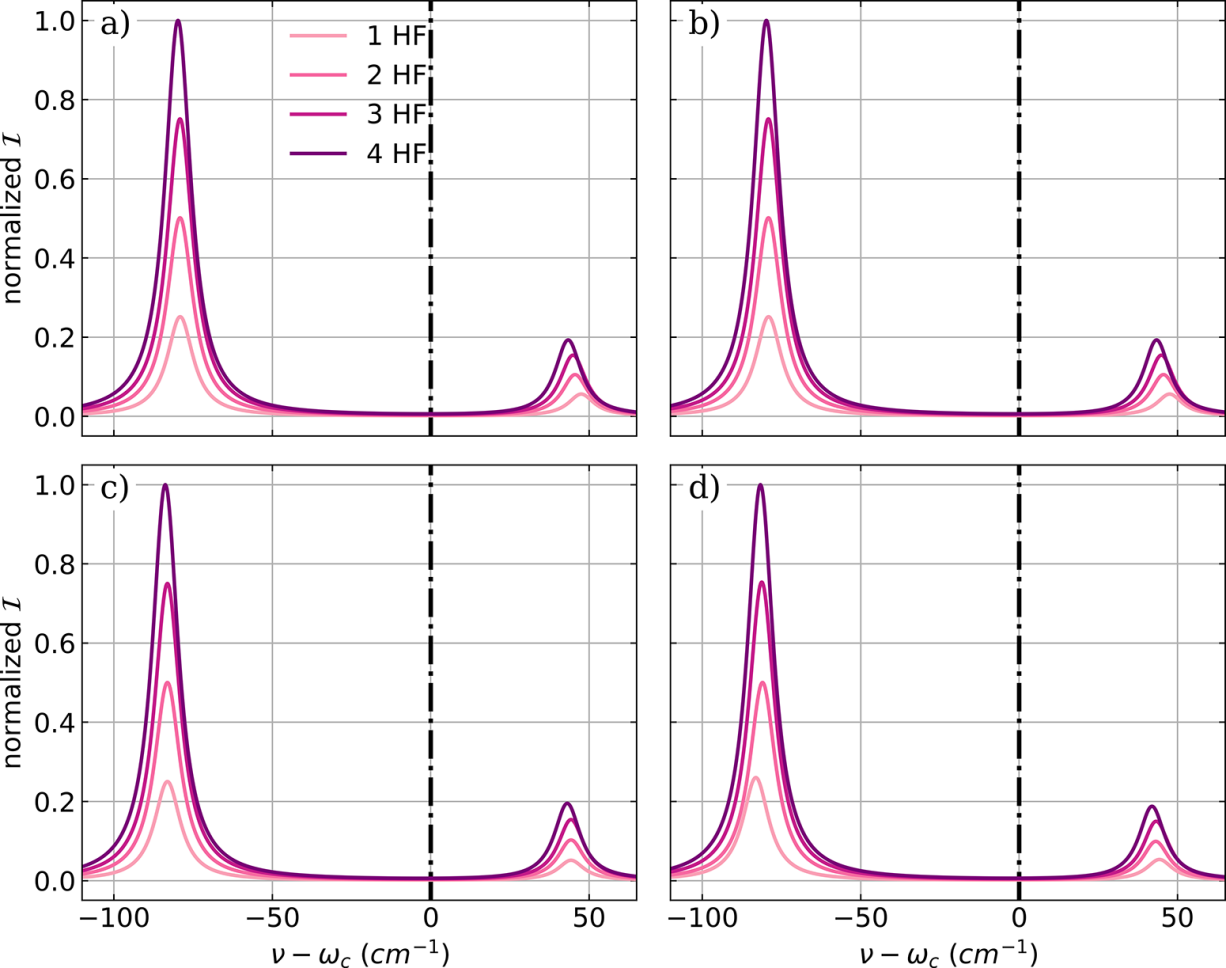
Vibro-polaritonic IR spectra calculated in the harmonic approximation for different numbers of HF molecules (color-coded) shown with respect to the cavity frequency ω_*c*_. The cavity is resonant with the harmonic fundamental transition (^*H*^ν_1_ = 4467 cm^–1^, black dashed-dotted lines) and a rescaled coupling strength of λ_0_ of 0.057 au is used (see eq 17). a) Full CBO-HF simulation in the *all-parallel* configuration. b) Full CBO-HF simulation in the *antiparallel* configuration. c) Linear CBO-HF simulation (without DSE terms) in the *all-parallel* configuration. c) Linear CBO-HF simulation (without DSE terms) in the *antiparallel* configuration.

**Figure 7:**
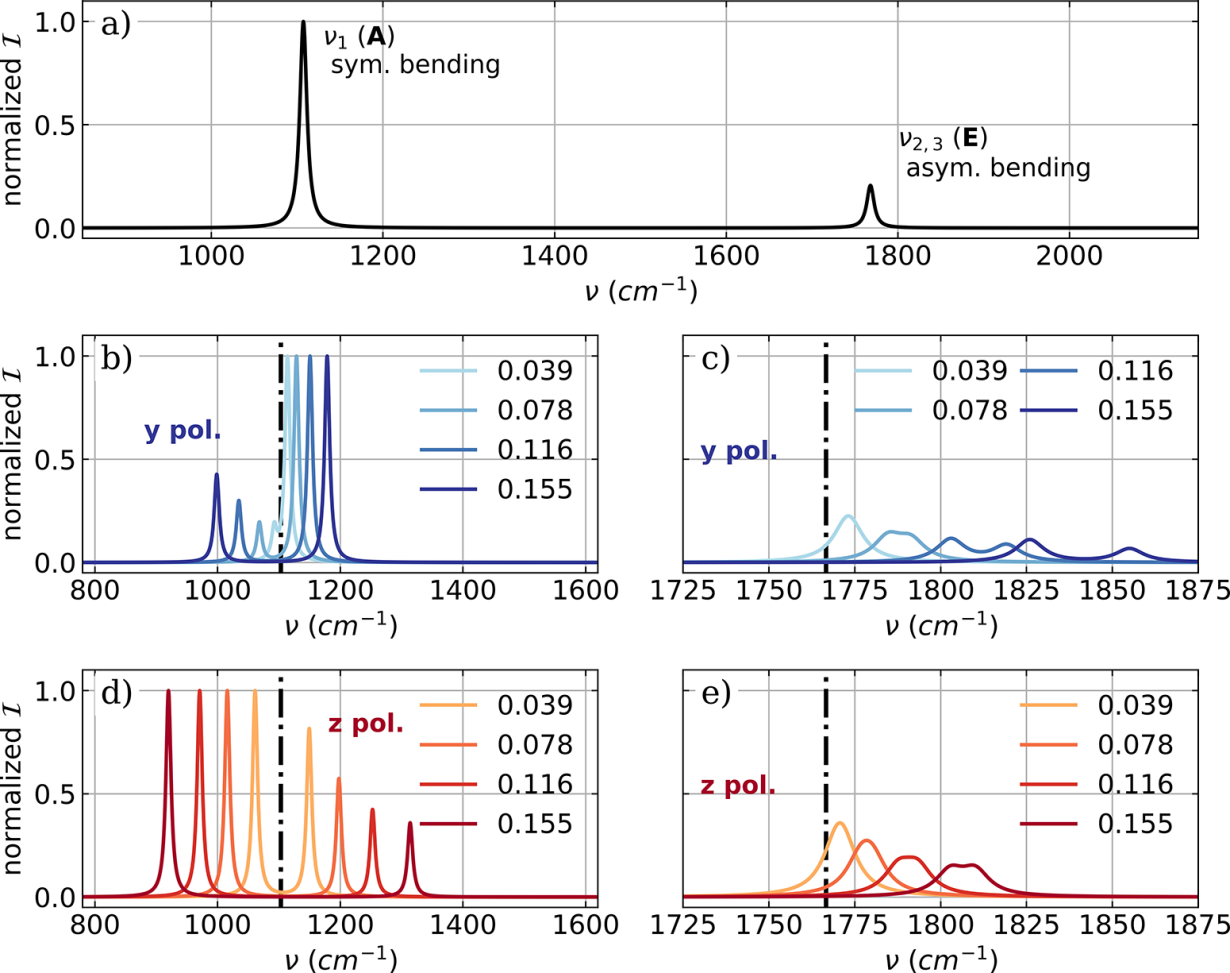
a) Vibronic IR spectra of a single NH_3_ molecule calculated in the harmonic approximation. The low energy part of the vibro-polaritonic IR spectra of a single NH_3_ molecule zoomed into the symmetric mode (b,d) and the two asymmetric bending modes shown in (c,e). The polarization axis of the cavity mode is the *y* axis for (b,c) and equal to the *z* axis for (d,e). The cavity frequency ω_*c*_ is resonant with the symmetric bending mode (1103 cm^–1^) and the cavity field strength λ_*c*_ increases from 0.039 au to 0.155 au.

## Supporting Information Available

Corrected Figures S3, S4, and S13 (PDF)

