# Supplementary material for: Ab Initio Vibro-Polaritonic Spectra in Strongly Coupled Cavity-Molecule Systems
Source: J Chem Theory Comput. 2023 Dec 12;19(24):9278–89. doi: 10.1021/acs.jctc.3c01135 (PMC10753771; doi:10.1021/acs.jctc.3c01135)
Supplement: Supplementary file 1 — ct3c01135_si_001.pdf [file ct3c01135_si_001.pdf]

Supporting Information: Ab-Initio Vibro-Polaritonic Spectra in Strongly Coupled Cavity-Molecule Systems

Thomas Schnappinger* and Markus Kowalewski*

Department of Physics, Stockholm University, AlbaNova University Center, SE-106 91 Stockholm, Sweden

E-mail: thomas.schnappinger@fysik.su.se; markus.kowalewski@fysik.su.se

Contents

S1 Derivation of the CBO-Hartree-Fock gradients **3**

S2 Validation of the Harmonic Approximation **7**

S3 Nuclear-Photonic Eigenfunctions vs Vibro-polaritonic Normal Modes **12**

S4 Beyond Diatomic Molecules: Vibro-Polaritonic Spectra of NH_3 **22**

References **24**

S1 Derivation of the CBO-Hartree-Fock gradients

The energy expectation value $\langle E_{CBO}\rangle$ in the basis of the atomic orbitals has the following form:

$$\begin{aligned}\langle E_{CBO}\rangle =& \sum_{\alpha,\beta}^{M} D_{\alpha,\beta}\left(\langle\alpha|\hat{h}|\beta\rangle + \omega_c q_c\langle\alpha|\hat{x}|\beta\rangle + X\langle\alpha|\hat{x}|\beta\rangle + \frac{1}{2}\langle\alpha|\hat{x}^2|\beta\rangle\right)\\ &+ \frac{1}{2}\sum_{\alpha,\beta,\gamma,\delta}^{M} D_{\alpha,\beta}D_{\gamma,\delta}\left(\langle\alpha\gamma|\hat{g}|\beta\delta\rangle + \langle\alpha|\hat{x}|\beta\rangle\langle\gamma|\hat{x}|\delta\rangle - \langle\alpha|\hat{x}|\delta\rangle\langle\gamma|\hat{x}|\beta\rangle\right)\\ &+ \tilde{E}_{n,c} \quad \text{with} \quad \tilde{E}_{n,c} = V_{nn} + \frac{1}{2}\omega_c^2 q_c^2 + E_{lin}^{(nuc)} + E_{dse}^{(nuc)}.\end{aligned} \tag{S1}$$

Where $\hat{x}$ and X are the electronic and nuclear part of the projected molecular dipole moment:

$$\hat{x} = -\boldsymbol{\lambda}_c \cdot \hat{\boldsymbol{r}} \;\text{ and }\; X = \boldsymbol{\lambda}_c \cdot \left(\sum_{A=1}^{N_{Nuc}} Z_A \boldsymbol{R}_A\right) \tag{S2}$$

The first derivative of the energy $\langle E_{CBO}\rangle$ with respect to a nuclear or photon displacement coordinate ζ_i is

$$\frac{\partial}{\partial\zeta_i}\langle E_{CBO}\rangle = \sum_{\alpha,\beta}^{M} D_{\alpha,\beta}\frac{\partial}{\partial\zeta_i}F_{\alpha,\beta} + \sum_{\alpha,\beta}^{M} F_{\alpha,\beta}\frac{\partial}{\partial\zeta_i}D_{\alpha,\beta} + \frac{\partial}{\partial\zeta_i}\tilde{E}_{n,c}. \tag{S3}$$

The first term in Eq. (S3) is the Hellmann-Feynman term, the second term is the wave-function derivative, or Pulay term, and the last part is the derivative of all scalar energy contributions. The full Hellmann-Feynman term has the following structure:

$$\begin{aligned}\sum_{\alpha,\beta}^{M} D_{\alpha,\beta}\frac{\partial}{\partial\zeta_i}F_{\alpha,\beta} =& \sum_{\alpha,\beta}^{M} D_{\alpha,\beta}\frac{\partial}{\partial\zeta_i}\left(\langle\alpha|\hat{h}|\beta\rangle + \omega_c q_c\langle\alpha|\hat{x}|\beta\rangle + X\langle\alpha|\hat{x}|\beta\rangle + \frac{1}{2}\langle\alpha|\hat{x}^2|\beta\rangle\right)\\ &+ \frac{1}{2}\sum_{\alpha,\beta,\gamma,\delta}^{M} D_{\alpha,\beta}D_{\gamma,\delta}\frac{\partial}{\partial\zeta_i}\left(\langle\alpha\gamma|\hat{g}|\beta\delta\rangle + \langle\alpha|\hat{x}|\beta\rangle\langle\gamma|\hat{x}|\delta\rangle - \langle\alpha|\hat{x}|\delta\rangle\langle\gamma|\hat{x}|\beta\rangle\right)\end{aligned} \tag{S4}$$

Since the cavity Born-Oppenheimer Hartree-Fock (CBO-HF) wave function is variational optimized, the explicit calculation of the density matrix derivatives can be avoided[1] and the Pulay term can be written in terms of overlap integral derivatives:

$$\begin{aligned}\sum_{\alpha,\beta}^{M} F_{\alpha,\beta}\frac{\partial D_{\alpha,\beta}}{\partial\zeta_i} &= -\sum_{\alpha,\beta}^{M} W_{\alpha,\beta}\frac{\partial S_{\alpha,\beta}}{\partial\zeta_i} \\ &= \sum_{\alpha,\beta}^{M}\sum_{i=1}^{N_{oc}} \epsilon_i c_{i,\alpha}^{*} c_{i,\beta}\left(\langle\frac{\partial\alpha}{\zeta_i}|\beta\rangle + \langle\alpha|\frac{\partial\beta}{\zeta_i}\rangle\right)\end{aligned} \tag{S5}$$

The remaining derivative is simpler since it dose not involve electron coordinates:

$$\frac{\partial}{\partial\zeta_i}\tilde{E}_{n,c} = \frac{\partial}{\partial\zeta_i}\left(V_{nn} + \frac{1}{2}\omega_c^2 q_c^2 + E_{lin}^{(nuc)} + E_{dse}^{(nuc)}\right) \tag{S6}$$

In the following two cases will be discussed, the first where ζ_i is a nuclear coordinate R_i and the second where ζ_i is a photon displacement coordinate q_c. For the first case, the nuclear derivatives of $\langle\hat{h}\rangle$, $\langle\hat{g}\rangle$ (in Eq. (S4)), V_{nn} (in Eq. (S6)) and the overlap integral derivatives in (in Eq. (S5)) are identical to the standard Hartree-Fock gradient terms and can be found in the literature.[1–4] Therefore, we focus on the new terms introduced by the CBO-HF ansatz in Eq. (S4) and Eq. (S6). The nuclear derivative of the linear cavity-electron interaction term takes the following form:

$$\begin{aligned}\frac{\partial}{\partial R_i}\omega_c q_c\langle\alpha|\hat{x}|\beta\rangle &= \omega_c q_c\left(\langle\frac{\partial\alpha}{\partial R_i}|\hat{x}|\beta\rangle + \langle\alpha|\frac{\partial\hat{x}}{\partial R_i}|\beta\rangle + \langle\alpha|\hat{x}|\frac{\partial\beta}{\partial R_i}\rangle\right) \\ &= \omega_c q_c\left(\langle\frac{\partial\alpha}{\partial R_i}|\hat{x}|\beta\rangle + \langle\alpha|\hat{x}|\frac{\partial\beta}{\partial R_i}\rangle\right)\end{aligned} \tag{S7}$$

Here the central or Hellmann-Feynman term becomes equal zero since $\hat{x}$ is independent of R_i. The nuclear derivatives of the pure electronic contributions to the dipole self-energy (DSE) follow a similar scheme. Thus, the one-electron contributions (Eq. (S8)) are simpler

than the two-electron contributions (Eq. (S9)).

$$\frac{1}{2}\frac{\partial}{\partial R_i}\langle\alpha|\hat{x}^2|\beta\rangle = \frac{1}{2}\left(\langle\frac{\partial\alpha}{\partial R_i}|\hat{x}^2|\beta\rangle + \langle\alpha|\hat{x}^2|\frac{\partial\beta}{\partial R_i}\rangle\right) \tag{S8}$$

$$\begin{aligned}\frac{1}{2}\frac{\partial}{\partial R_i}\langle\alpha|\hat{x}|\beta\rangle\langle\gamma|\hat{x}|\delta\rangle &= \frac{1}{2}\left(\frac{\partial}{\partial R_i}\langle\alpha|\hat{x}|\beta\rangle\right)\langle\gamma|\hat{x}|\delta\rangle + \frac{1}{2}\langle\alpha|\hat{x}|\beta\rangle\left(\frac{\partial}{\partial R_i}\langle\gamma|\hat{x}|\delta\rangle\right)\\ &= \frac{1}{2}\left(\langle\frac{\partial\alpha}{\partial R_i}|\hat{x}|\beta\rangle + \langle\alpha|\hat{x}|\frac{\partial\beta}{\partial R_i}\rangle\right)\langle\gamma|\hat{x}|\delta\rangle + \frac{1}{2}\langle\alpha|\hat{x}|\beta\rangle\left(\langle\frac{\partial\gamma}{\partial R_i}|\hat{x}|\delta\rangle + \langle\gamma|\hat{x}|\frac{\partial\delta}{\partial R_i}\rangle\right)\end{aligned} \tag{S9}$$

The nuclear derivative for the mixed electron-nuclear DSE is

$$\begin{aligned}\frac{\partial}{\partial R_i}X\langle\alpha|\hat{x}|\beta\rangle &= \left(\frac{\partial}{\partial R_i}X\right)\langle\alpha|\hat{x}|\beta\rangle + X\left(\frac{\partial}{\partial R_i}\langle\alpha|\hat{x}|\beta\rangle\right)\\ &= \left(\frac{\partial}{\partial R_i}\boldsymbol{\lambda}_c\cdot\left(\sum_{A=1}^{N_{Nuc}} Z_A\boldsymbol{R}_A\right)\right)\langle\alpha|\hat{x}|\beta\rangle + X\left(\langle\frac{\partial\alpha}{\partial R_i}|\hat{x}|\beta\rangle + \langle\alpha|\hat{x}|\frac{\partial\beta}{\partial R_i}\rangle\right)\\ &= \lambda_i Z_i\langle\alpha|\hat{x}|\beta\rangle + X\left(\langle\frac{\partial\alpha}{\partial R_i}|\hat{x}|\beta\rangle + \langle\alpha|\hat{x}|\frac{\partial\beta}{\partial R_i}\rangle\right)\end{aligned} \tag{S10}$$

Regarding Eq. (S6) there are only two "new" nuclear derivatives:

$$\frac{\partial}{\partial R_i}E_{lin}^{(nuc)} = \frac{\partial}{\partial R_i}\omega_c q_c X = \frac{\partial}{\partial R_i}\omega_c q_c\boldsymbol{\lambda}_c\cdot\left(\sum_{A=1}^{N_{Nuc}} Z_A\boldsymbol{R}_A\right) = \omega_c q_c\lambda_i Z_i \tag{S11}$$

$$\frac{\partial}{\partial R_i}E_{dse}^{(nuc)} = \frac{1}{2}\frac{\partial}{\partial R_i}\left(\boldsymbol{\lambda}_c\cdot\boldsymbol{\mu}_{Nuc}\right)^2 = \frac{1}{2}\frac{\partial}{\partial R_i}\left(\boldsymbol{\lambda}_c\cdot\left(\sum_{A=1}^{N_{Nuc}} Z_A\boldsymbol{R}_A\right)\right)^2 \tag{S12}$$

For the second case ζ_i being a photon displacement coordinate q_c the Pulay term in Eq. (S5) is zero, since the atomic orbitals used are independent of q_c. This is also the reason why for the Hellmann-Feynman part of the derivative (Eq. (S4)) only terms that explicitly depend on q_c are of relevance. In combination with relevant parts of Eq. (S6) the following

expressions for the derivative with respect to q_c is found:

$$\begin{aligned}\frac{\partial}{\partial q_c} E_{CBO} =& \omega_c^2 q_c + \omega_c \sum_{\alpha,\beta}^{M} D_{\alpha,\beta} \langle \alpha | \hat{x} | \beta \rangle + \omega_c \boldsymbol{\lambda}_c \cdot \boldsymbol{\mu}_{Nuc} \\ =& \omega_c^2 q_c - \omega_c \left(\boldsymbol{\lambda}_c \cdot \langle \hat{\boldsymbol{\mu}} \rangle \right)\end{aligned} \tag{S13}$$

This equivalent to the result obtained using the Hellmann–Feynman theorem.[5,6]

S2 Validation of the Harmonic Approximation

The spectra of an individual HF molecule without coupling to an optical cavity calculated in the harmonic approximation and full quantum mechanical (anharmonic) are shown in Fig. S1. The fundamental vibrational transition in the harmonic approximation has a frequency of ${}^{H}\nu_1 = 4467\,\mathrm{cm}^{-1}$. The fully quantum mechanical treatment leads to a strong red-shifted of $186\,\mathrm{cm}^{-1}$ for the fundamental vibrational transition (${}^{A}\nu_1 = 4281\,\mathrm{cm}^{-1}$).

The vibro-polaritonic infrared spectra for different coupling strengths calculated in the harmonic approximation and full-quantum (anharmonic) as well as the trends in the Rabi splitting frequency Ω_R are shown for the *all-parallel* configuration of two HF molecules in Fig. S2 and for the *antiparallel* configuration of two HF molecules in Fig. S3. Due to the rescaling of $\boldsymbol{\lambda}_c$ the observed spectra are nearly identical to the ones obtained for a single HF molecule. Consistent with our findings on the properties of ensembles of molecules coupled to an optical cavity,[6] the vibro-polaritonic IR spectra are nearly indistinguishable for the two configurations studied. The only noticeable difference is the asymmetry of the Rabi splitting $\Delta\Omega_R = \omega_c - 0.5\left(\nu^{LP} + \nu^{UP}\right)$ shown in Fig. S2 c) and Fig. S3 c). The value of $\Delta\Omega_R$ is slightly smaller for the *antiparallel* configuration. Fig. S4 shows the difference between Ω_R and $\Delta = \omega_c -^{H} \nu_1$ in the harmonic approximation, as well as the cavity-induced energy changes E_{lin}, E_{dse} and E_{dis} as a function of the cavity frequency ω_c, keeping λ_c fixed for both configurations. Also, for the bimolecular case, the largest Rabi splitting Ω_R is obtained for ω_c resonant with the fundamental molecular transition (${}^{H}\nu_1$). Consistent with the single-molecule results, the difference between Ω_R and the detuning Δ is not symmetric with respect to ${}^{H}\nu_1$ and tends to a finite nonzero value even for large detunings, see Fig. S4 a). Regarding the resonant spectra (Fig. S2 and Fig. S3), the effect of cavity detuning is the same for both configurations; see gray line Fig. S4 a). The cavity-induced energy modifications E_{lin}, E_{dse} and E_{dis} shown in Fig. S4 b) for the *all-parallel* configuration and in Fig. S4 c) for the *antiparallel* configuration are different for each configuration but are constant for all values of ω_c.

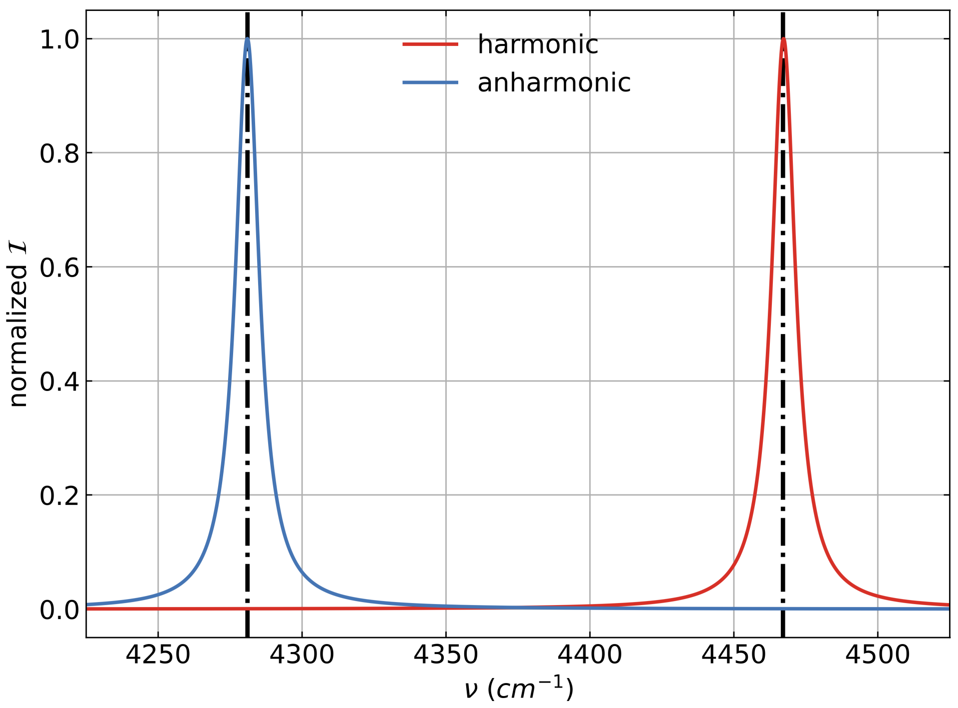

Figure S1: Vibronic IR spectra of a single HF molecule calculated in the harmonic approximation (red) and the full anharmonic simulation (blue). Black dashed-dotted lines indicate the frequencies of the harmonic ($4467\,\mathrm{cm}^{-1}$) and anharmonic ($4281\,\mathrm{cm}^{-1}$) fundamental transitions.

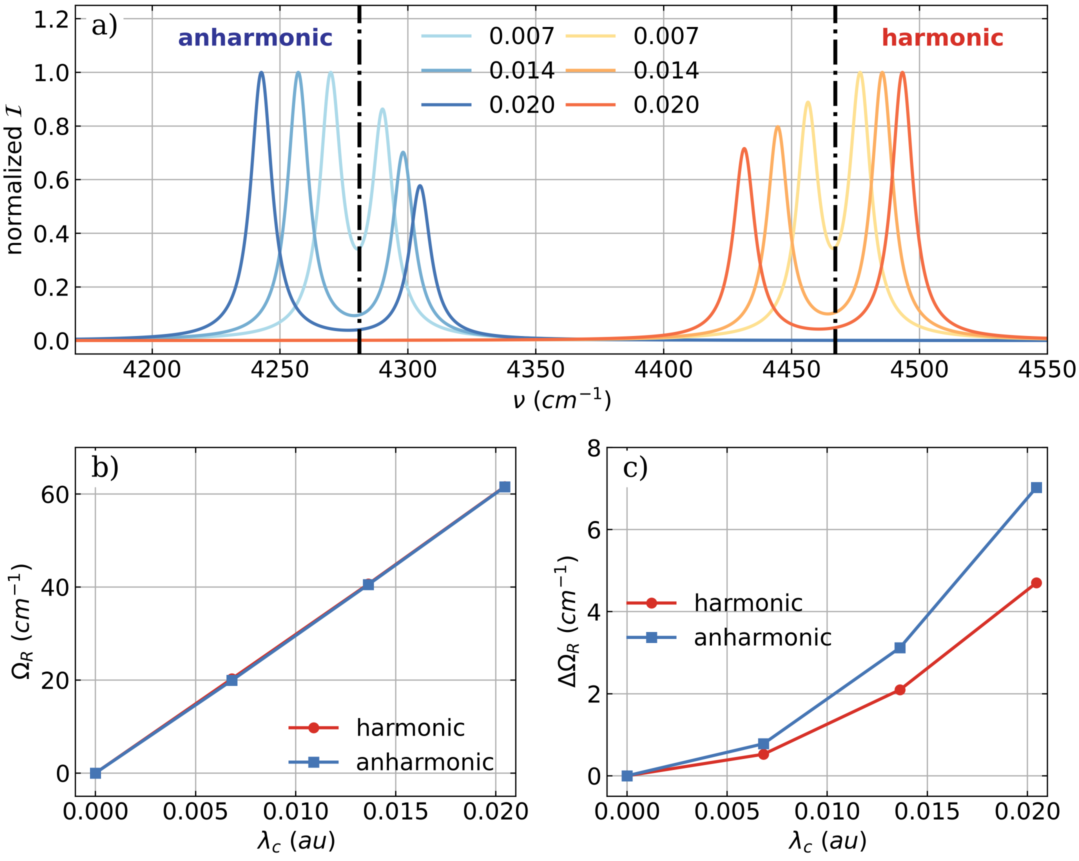

Figure S2: a) Vibro-polaritonic IR spectra of a two parallel HF molecule calculated in the harmonic approximation (reddish) and in the full anharmonic setup (bluish). Black dashed-dotted lines indicate the frequencies of the harmonic ($4467\,\mathrm{cm}^{-1}$) and anharmonic ($4281\,\mathrm{cm}^{-1}$) fundamental transitions. The cavity frequency ω_c is resonant with the corresponding fundamental transition in both cases, and the coupling strength λ_c is increased from 0.009 au to 0.039 au (from lightest to darkest color). b) Rabi splitting Ω_R as a function of λ_c. c) Asymmetry $\Delta\Omega_R = \omega_c - 0.5\left(\nu^{LP} + \nu^{UP}\right)$ of the Rabi splitting.

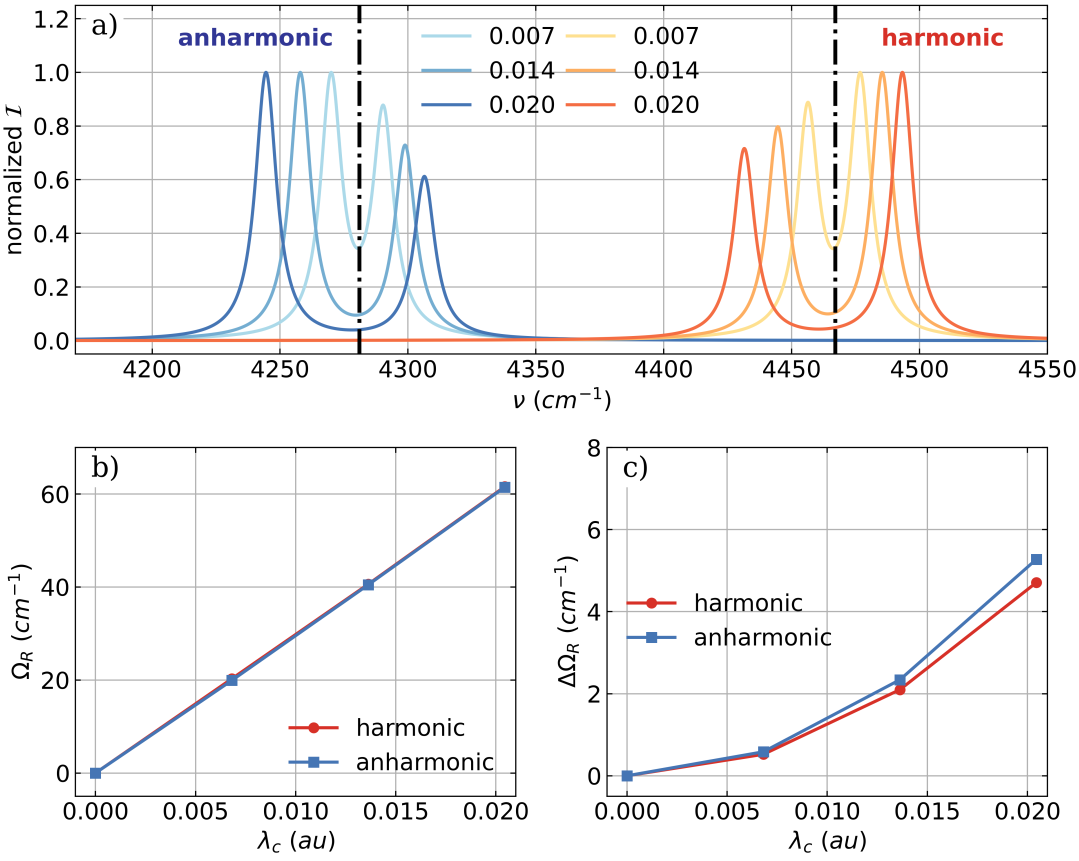

Figure S3: a) Vibro-polaritonic IR spectra of a two antiparallel HF molecules calculated in the harmonic approximation (reddish) and in the full anharmonic setup (bluish). Black dashed-dotted lines indicate the frequencies of the harmonic ($4467\,\mathrm{cm}^{-1}$) and anharmonic ($4281\,\mathrm{cm}^{-1}$) fundamental transitions. The cavity frequency ω_c is resonant with the corresponding fundamental transition in both cases, and the coupling strength λ_c is increased from $0.009\,\mathrm{au}$ to $0.039\,\mathrm{au}$ (from lightest to darkest color). b) Rabi splitting Ω_R as a function of λ_c. c) Asymmetry $\Delta\Omega_R = \omega_c - 0.5\left(\nu^{LP} + \nu^{UP}\right)$ of the Rabi splitting.

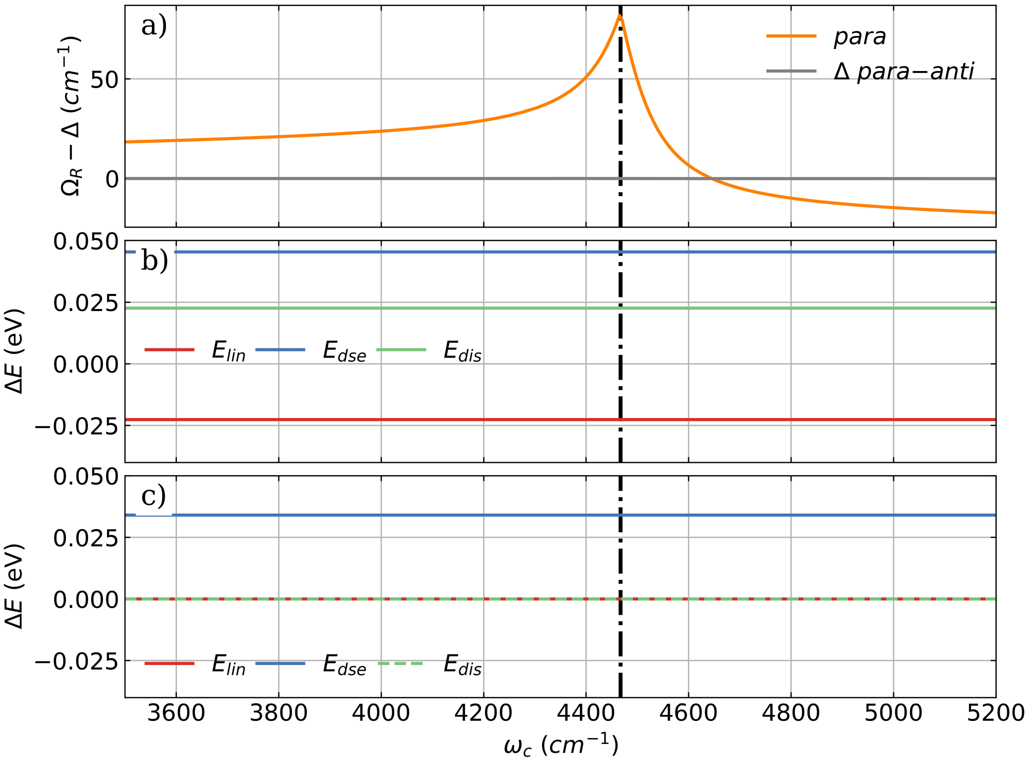

Figure S4: a) Difference between the Rabi splitting Ω_R and $\Delta = \omega_c -^H \nu_1$ as a function of the cavity frequency ω_c for two parallel aligned HF molecules. Here Δ describes the difference between the cavity frequency ω_c and the fundamental bare molecular transition ${}^H\nu_1$. The difference of $\Omega_R - \Delta$ between the *all-parallel* and the *antiparallel* configuration is shown in gray. Energy contributions due to the cavity interaction as function of the cavity frequency ω_c for b) the *all-parallel* orientation and c) the *antiparallel* configuration. Black dashed-dotted lines indicate the frequencies of the harmonic ($4467\,\mathrm{cm}^{-1}$) fundamental transition. A constant coupling strength λ_c of $0.02\,\mathrm{au}$ is used.

S3 Nuclear-Photonic Eigenfunctions vs Vibro-polaritonic Normal Modes

In this section, we compare the vibro-polaritonic normal mode vectors $\boldsymbol{a}_i$ obtained in the harmonic approximation with the full quantum calculated nuclear-photonic eigenfunctions χ for the case of a single HF molecule and for two parallel oriented HF molecules. Before we start the direct comparison, we need to define how to visualize both the normal mode and the eigenfunctions. Since HF is a diatomic molecule and the molecular bond is aligned with the z axis of the laboratory frame, we can represent the normal mode vectors $\boldsymbol{a}_i$ of size $(3N_A + 1)$ in a two-dimensional subspace spanned by the z axis of the laboratory frame, which includes both the z components of the H atom(s) and the F atom(s) and the photon displacement coordinate q_c. The nuclear-photonic eigenfunctions are represented as probability densities. For the single-molecule case, the densities are plotted in the full two-dimensional space spanned by the internal (bond length) coordinate R and the photon displacement coordinate q_c. To visualize the densities for the two HF molecules, all possible two-dimensional projections of the full three-dimensional space spanned by the two internal coordinates (R_1 and R_2) and q_c are used.

In Fig. S5 both the nuclear-photonic eigenfunctions and the vibro-polaritonic normal modes for a single HF molecule and a single cavity mode are shown. The cavity frequency ω_c is resonant with the corresponding fundamental transition (harmonic $4467\,\mathrm{cm}^{-1}$ and anharmonic $4281\,\mathrm{cm}^{-1}$) but the coupling strength λ_c is set to zero. The to-be lower polariton (LP) eigenfunction Fig. S5 c) is a pure one-photon cavity vacuum state with a nodal plan orthogonal to the q_c coordinate and the to-be upper polariton (UP) eigenfunction Fig. S5 e) is the pure first excited molecular vibrational eigenstate with a nodal plan orthogonal to the R coordinate. The vibro-polaritonic normal modes give the classical pendants. The to-be LP normal mode (reduced normal mode shown in Fig. S5 d)) has only a contribution along the photon displacement coordinate q_c, for visualization purposes shown on both atoms. The

to-be UP normal mode (reduced normal mode shown in Fig. S5 f)) has only a contribution along the z coordinate axis in the laboratory frame, which describes the stretching mode of the HF molecule. Since we are discussing mass-weighted normal modes, the vector describing the stretching mode, shown in Fig. S5 f), is predominantly localized on the hydrogen atom. The eigenfunctions and normal modes in the case of a single molecule for a coupling strength λ_c of 0.019 au are shown in Fig. S5. In the full-quantum eigenfunction picture (see Figs S6 c) and e)) the light-matter interaction manifests itself through a rotation of the probability densities in the q_c–R-plane accompanied by a slight deformation. As discussed in the manuscript (see Eq. 18) and in the literature[7] the eigenfunctions of the LP state and the UP state can be interpreted as linear combinations of the uncoupled one-photon cavity vacuum state and the first excited molecular vibrational state. The mixed characters of the LP state and UP state are also visible in the normal mode representation (see Fig S6 d) and f)). The reduced vectors in the coupled situation are linear combinations of the uncoupled vectors (Fig. S5 d) and f)). When analyzing not only the direction of the LP vector and the UP vector, but also their length, another effect of the cavity interaction is observed. For both normal modes in the coupled system, the mixing of light and matter leads to the situation that both vectors have significant components on both the H and F atoms, in contrast to the uncoupled case, see Fig. S5 f).

For the two parallel aligned HF molecules nuclear-photonic eigenfunctions and vibro-polaritonic normal modes for the LP state, the dark middle-polariton state and the UP state are shown in Fig. S7, Fig. S8, Fig. S9 for the uncoupled system ($\lambda_c = 0.0$) and in Fig. S10, Fig. S11, Fig. S12 for a coupling strength λ_c of 0.019 au. For the LP and UP states, identical behavior is observed when coupled to a cavity compared to the case of a single HF. In the uncoupled system, the second vibrational transition is dark and remains dark when coupled to a cavity. Therefore, the underlying eigenfunctions and normal modes remain unchanged; see (Fig. S8) and (Fig. S11).

Overall, both descriptions, the nuclear-photonic eigenfunctions and the vibro-polaritonic

normal modes, contain similar information about the formation of hybrid-light matter states.

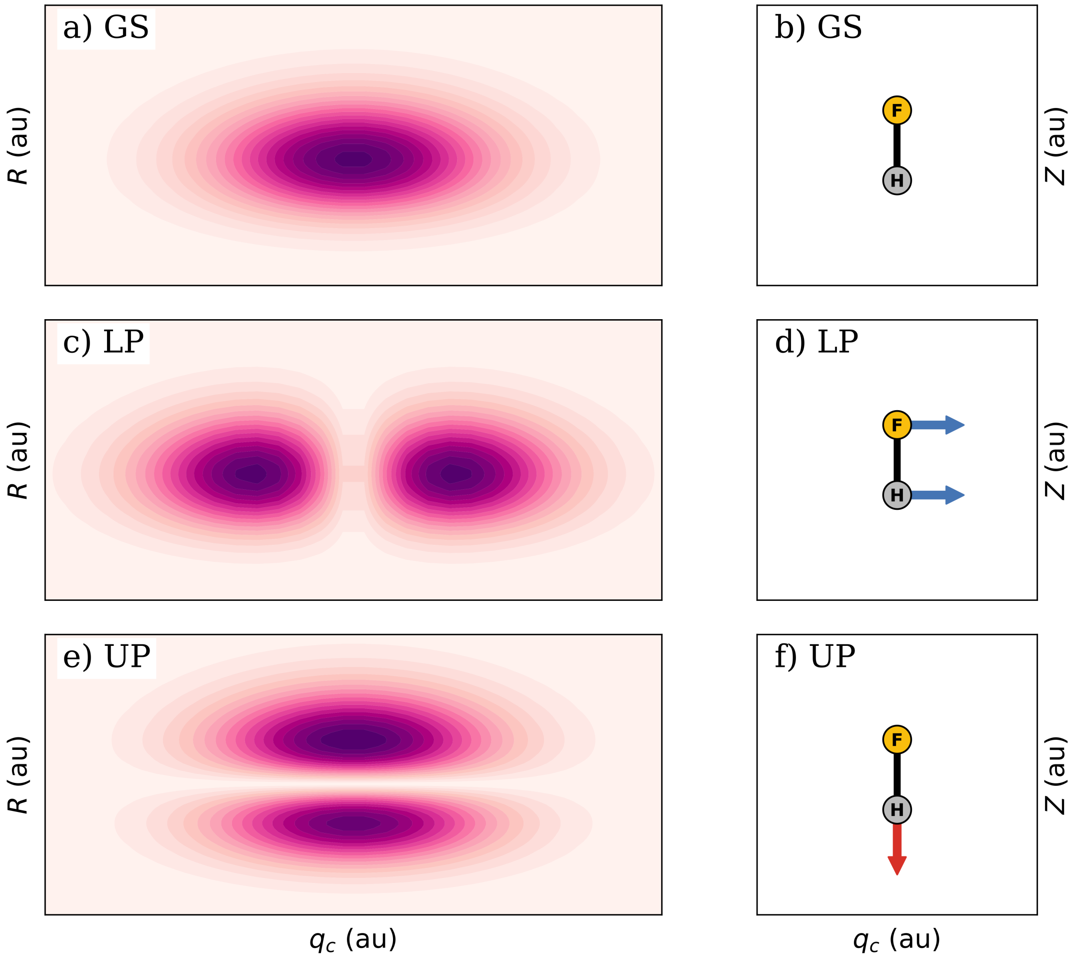

Figure S5: a), c), and d) first three eigenfunctions for a single HF molecule described on two-dimensional CBO-HF surface. b) classic nuclear configuration, d), and e) both mass-weighted normal modes of a single HF molecule in harmonic approximation. The cavity frequency ω_c is resonant with the corresponding fundamental transition (harmonic 4467 cm^{-1} and anharmonic 4281 cm^{-1}). The coupling strength λ_c is set to zero.

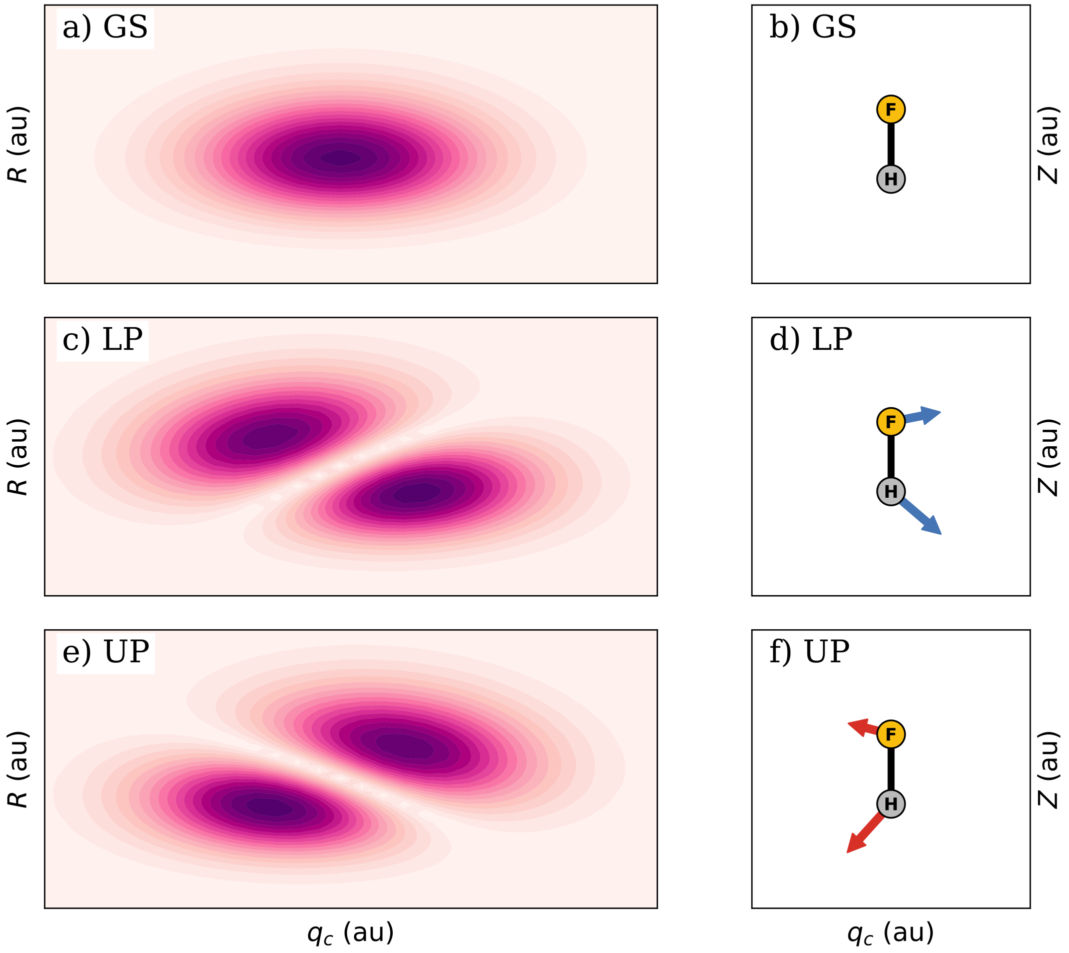

Figure S6: a), c), and d) first three eigenfunctions for a single HF molecule described on two-dimensional CBO-HF surface. b) classic nuclear configuration, d), and e) both both mass-weighted normal modes of a single HF molecule in harmonic approximation. The cavity frequency ω_c is resonant with the corresponding fundamental transition (harmonic $4467\,\mathrm{cm}^{-1}$ and anharmonic $4281\,\mathrm{cm}^{-1}$). The coupling strength λ_c is set to $0.019\,\mathrm{au}$.

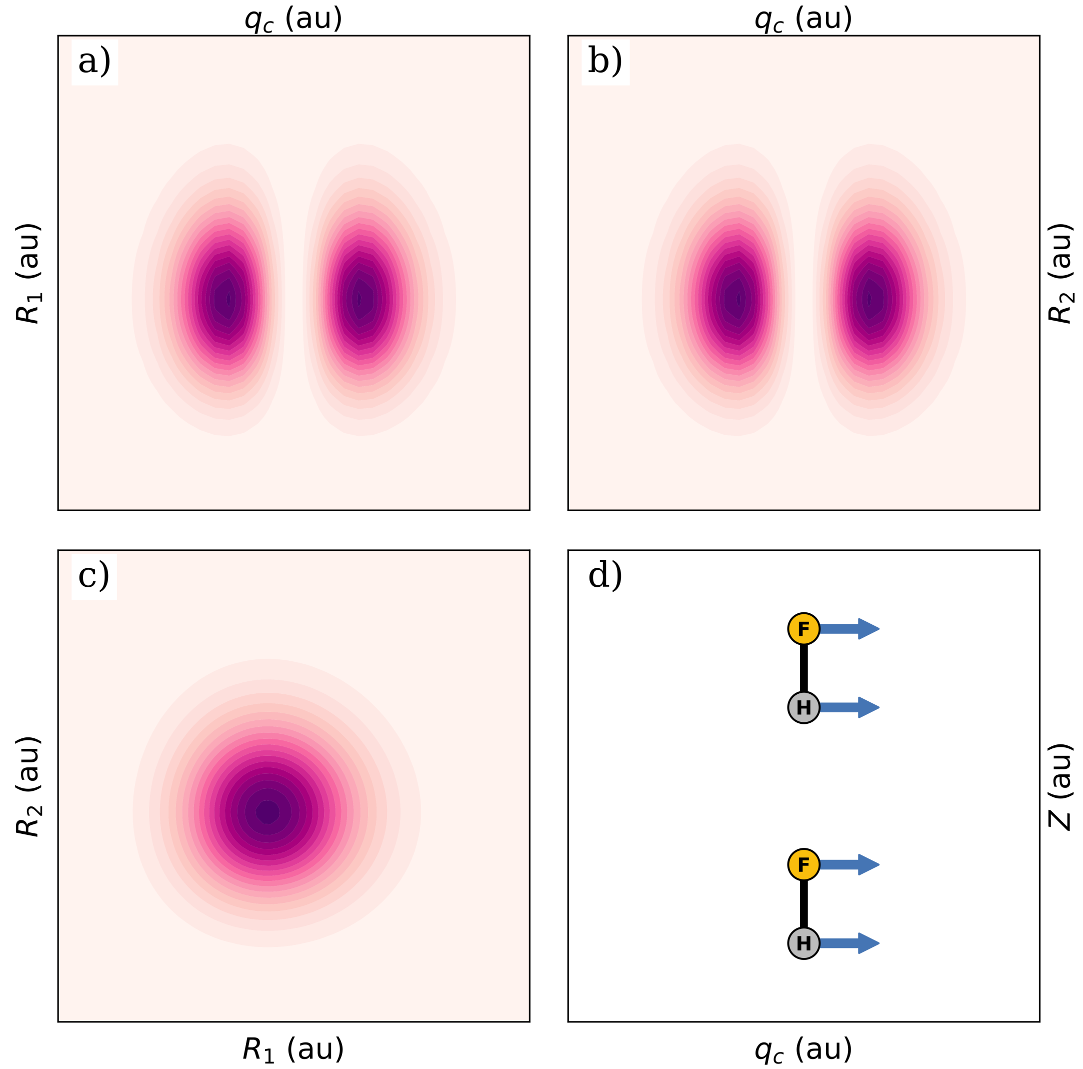

Figure S7: First excited state eigenfunction for two parallel HF molecules described on a three-dimensional CBO-HF surface. For a), b), and c, the eigenfunction is integrated over one coordinate each. d) classic nuclear configuration and first mass-weighted normal mode of two parallel HF molecules. The molecules are separated by a distance of 800 Å. The cavity frequency ω_c is resonant with the corresponding fundamental transition (harmonic $4467\,\mathrm{cm}^{-1}$ and anharmonic $4281\,\mathrm{cm}^{-1}$). The coupling strength λ_c is set to zero.

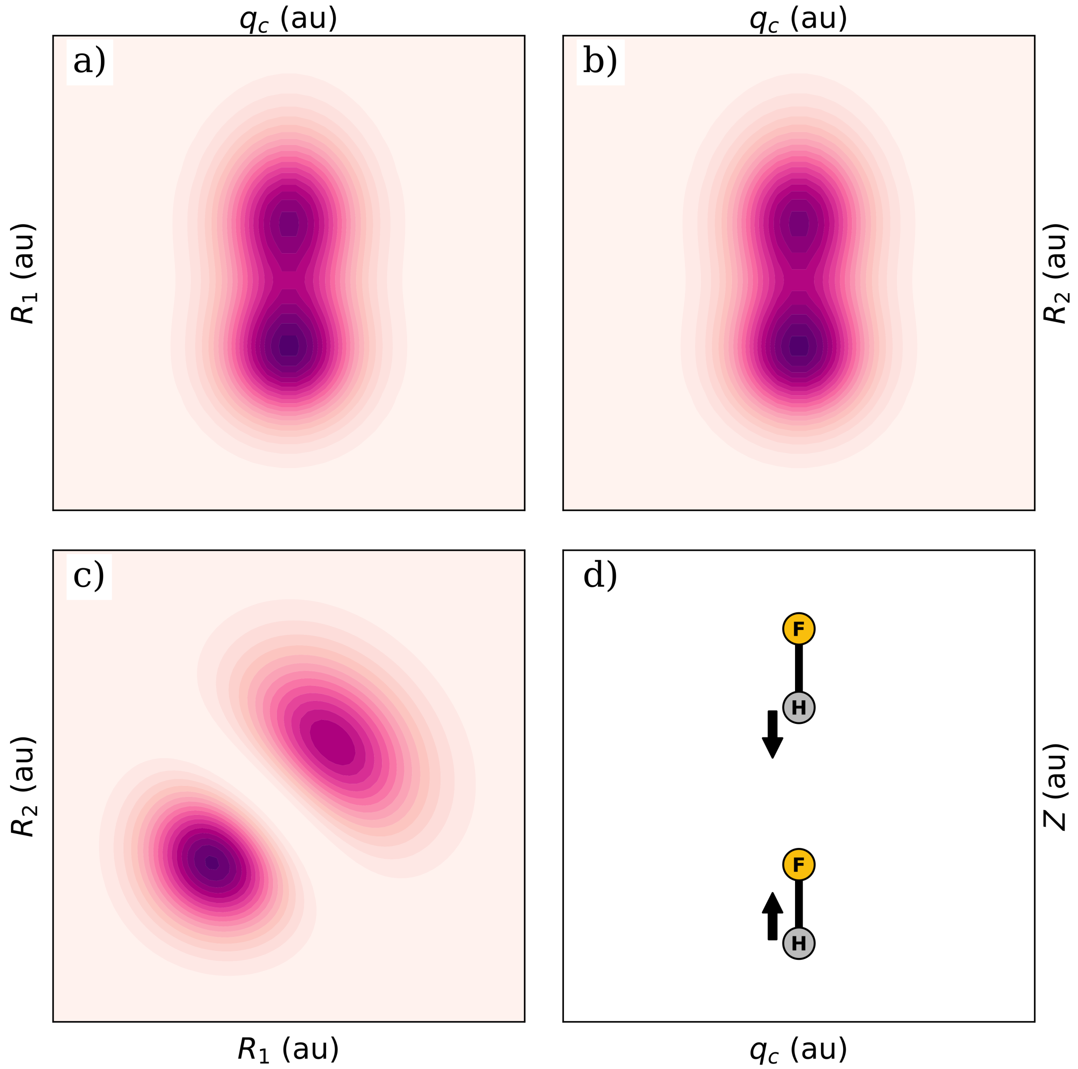

Figure S8: Second excited state eigenfunction for two parallel HF molecules described on a three-dimensional CBO-HF surface. For a), b), and c, the eigenfunction is integrated over one coordinate each. d) classic nuclear configuration and second mass-weighted normal mode of two parallel HF molecules. The molecules are separated by a distance of 800 Å. The cavity frequency ω_c is resonant with the corresponding fundamental transition (harmonic $4467\,\mathrm{cm}^{-1}$ and anharmonic $4281\,\mathrm{cm}^{-1}$). The coupling strength λ_c is set to zero.

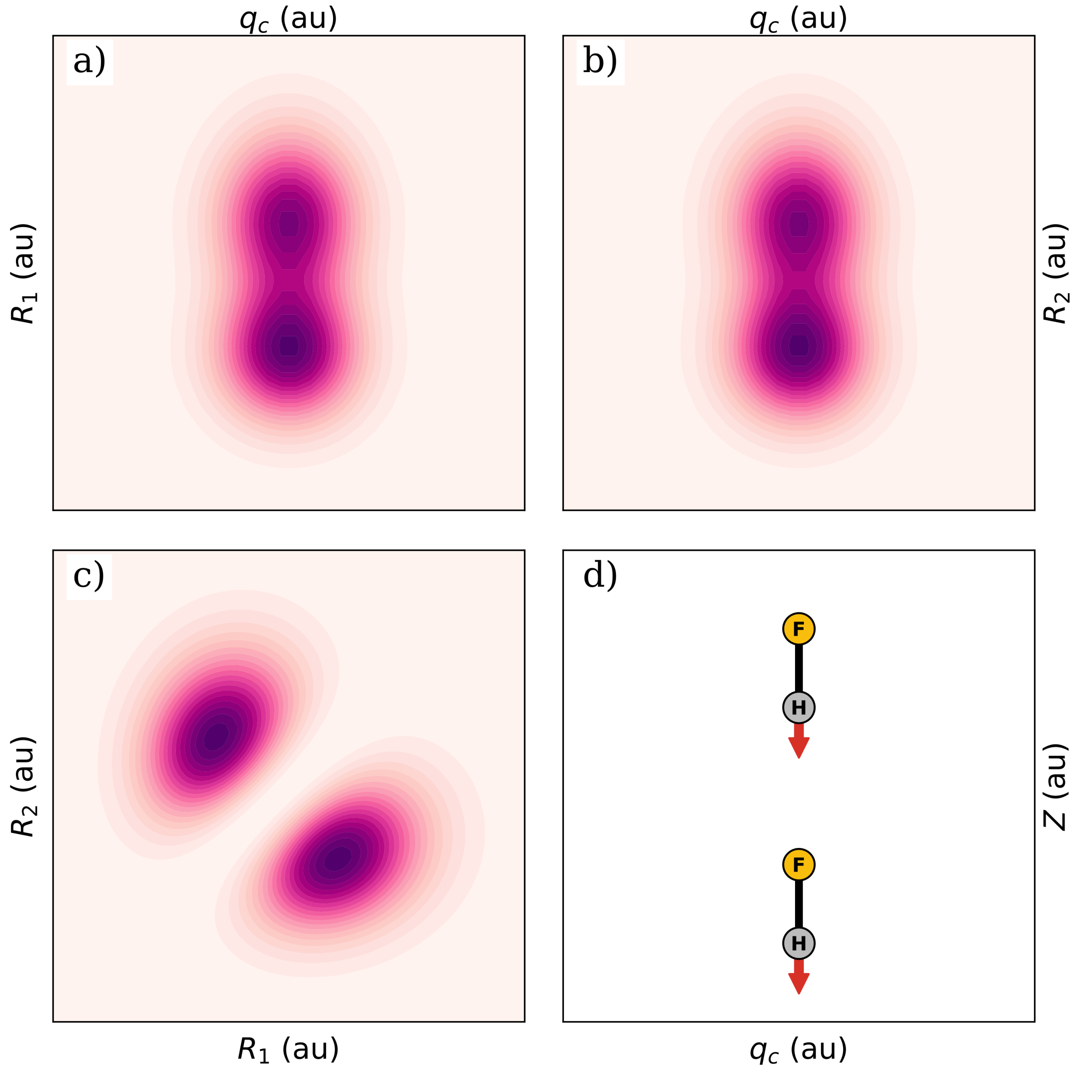

Figure S9: Third excited state eigenfunction for two parallel HF molecules described on a three-dimensional CBO-HF surface. For a), b), and c, the eigenfunction is integrated over one coordinate each. d) classic nuclear configuration and third mass-weighted normal mode of two parallel HF molecules. The molecules are separated by a distance of 800 Å. The cavity frequency ω_c is resonant with the corresponding fundamental transition (harmonic $4467\,\text{cm}^{-1}$ and anharmonic $4281\,\text{cm}^{-1}$). The coupling strength λ_c is set to zero.

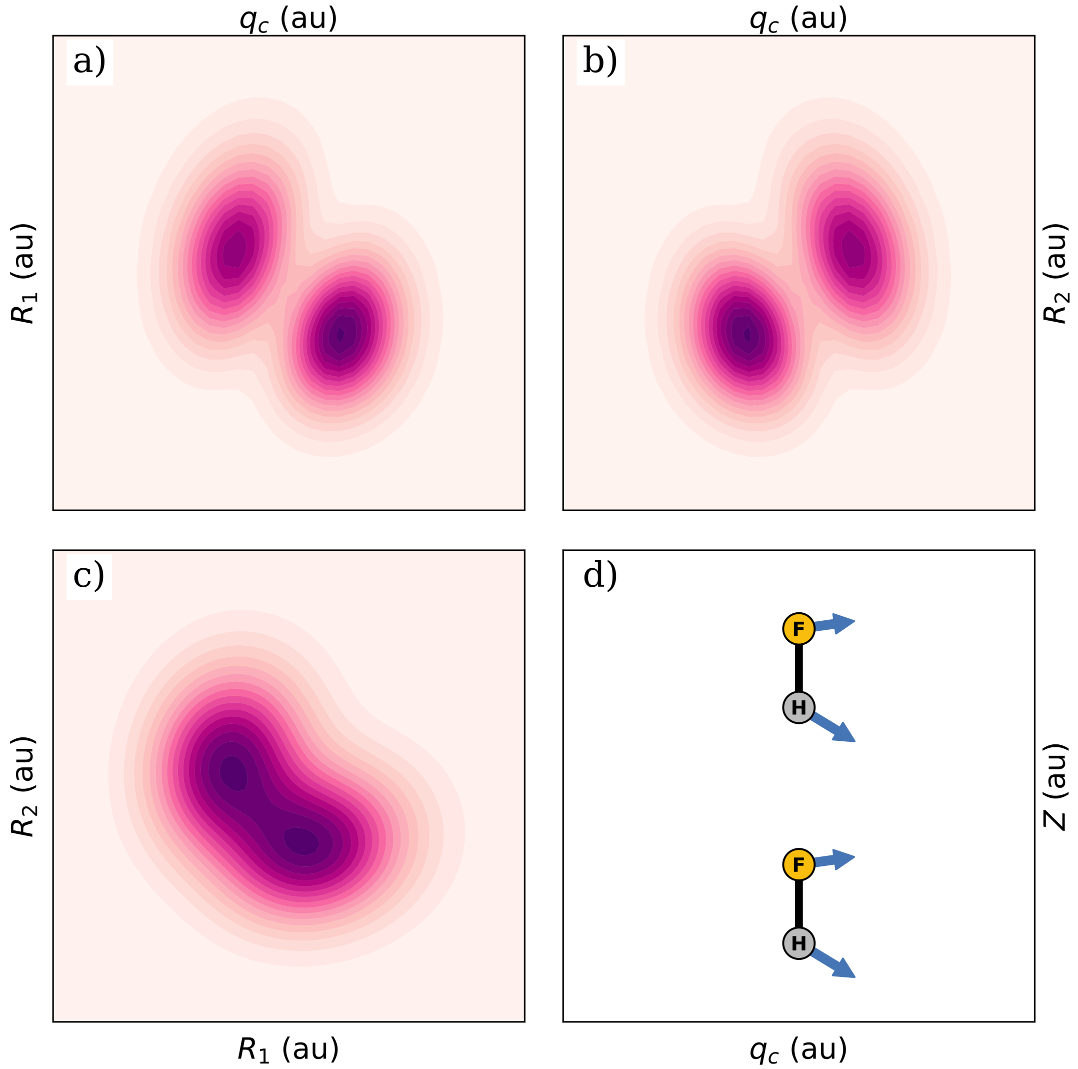

Figure S10: First excited state eigenfunction for two parallel HF molecules described on a three-dimensional CBO-HF surface. For a), b), and c, the eigenfunction is integrated over one coordinate each. d) classic nuclear configuration and first mass-weighted normal mode of two parallel HF molecules. The molecules are separated by a distance of 800 Å. The cavity frequency ω_c is resonant with the corresponding fundamental transition (harmonic $4467\,\mathrm{cm}^{-1}$ and anharmonic $4281\,\mathrm{cm}^{-1}$). The coupling strength λ_c is set to 0.019 au.

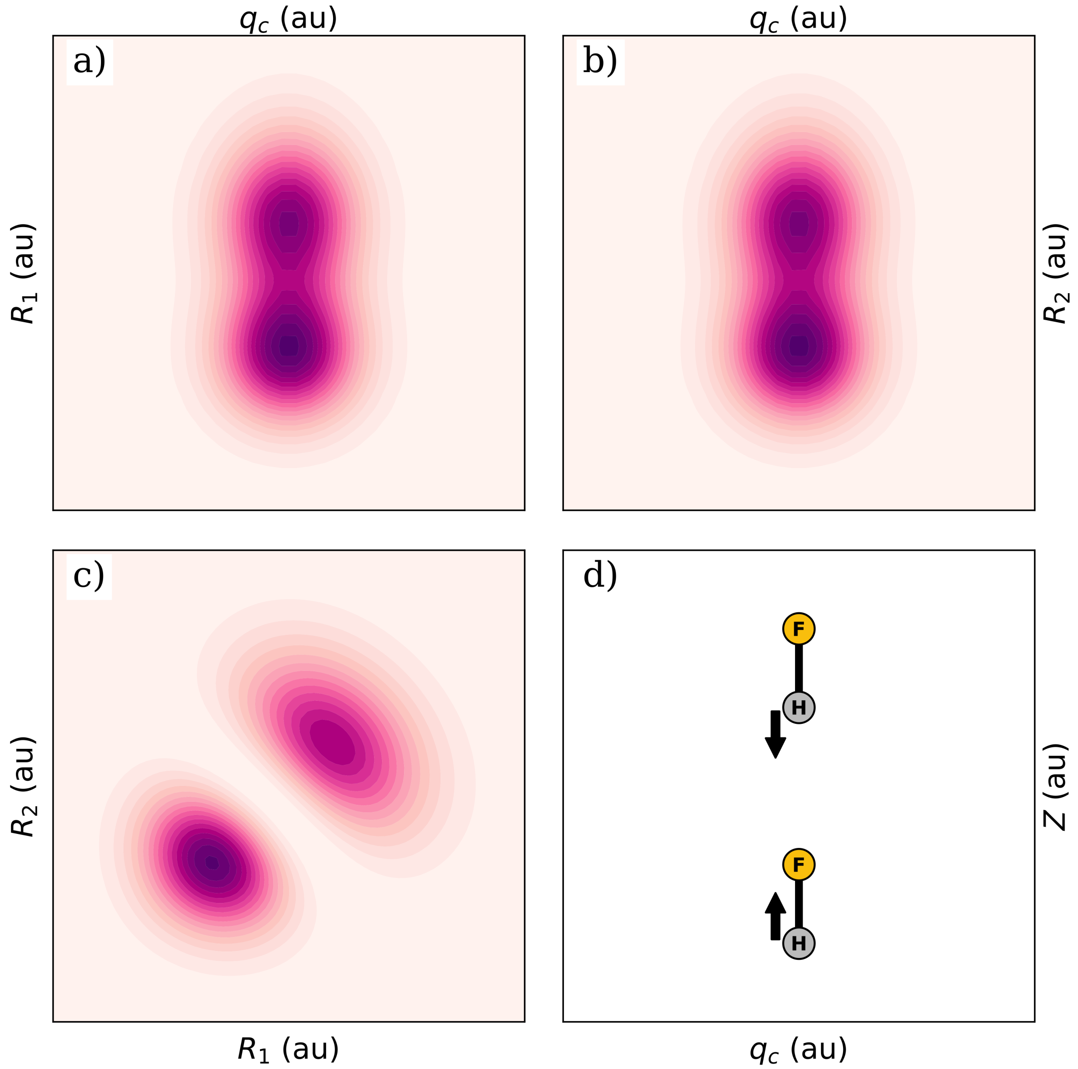

Figure S11: Second excited state eigenfunction for two parallel HF molecules described on a three-dimensional CBO-HF surface. For a), b), and c, the eigenfunction is integrated over one coordinate each. d) classic nuclear configuration and second mass-weighted normal mode of two parallel HF molecules. The molecules are separated by a distance of 800 Å. The cavity frequency ω_c is resonant with the corresponding fundamental transition (harmonic $4467\,\mathrm{cm}^{-1}$ and anharmonic $4281\,\mathrm{cm}^{-1}$). The coupling strength λ_c is set to 0.019 au.

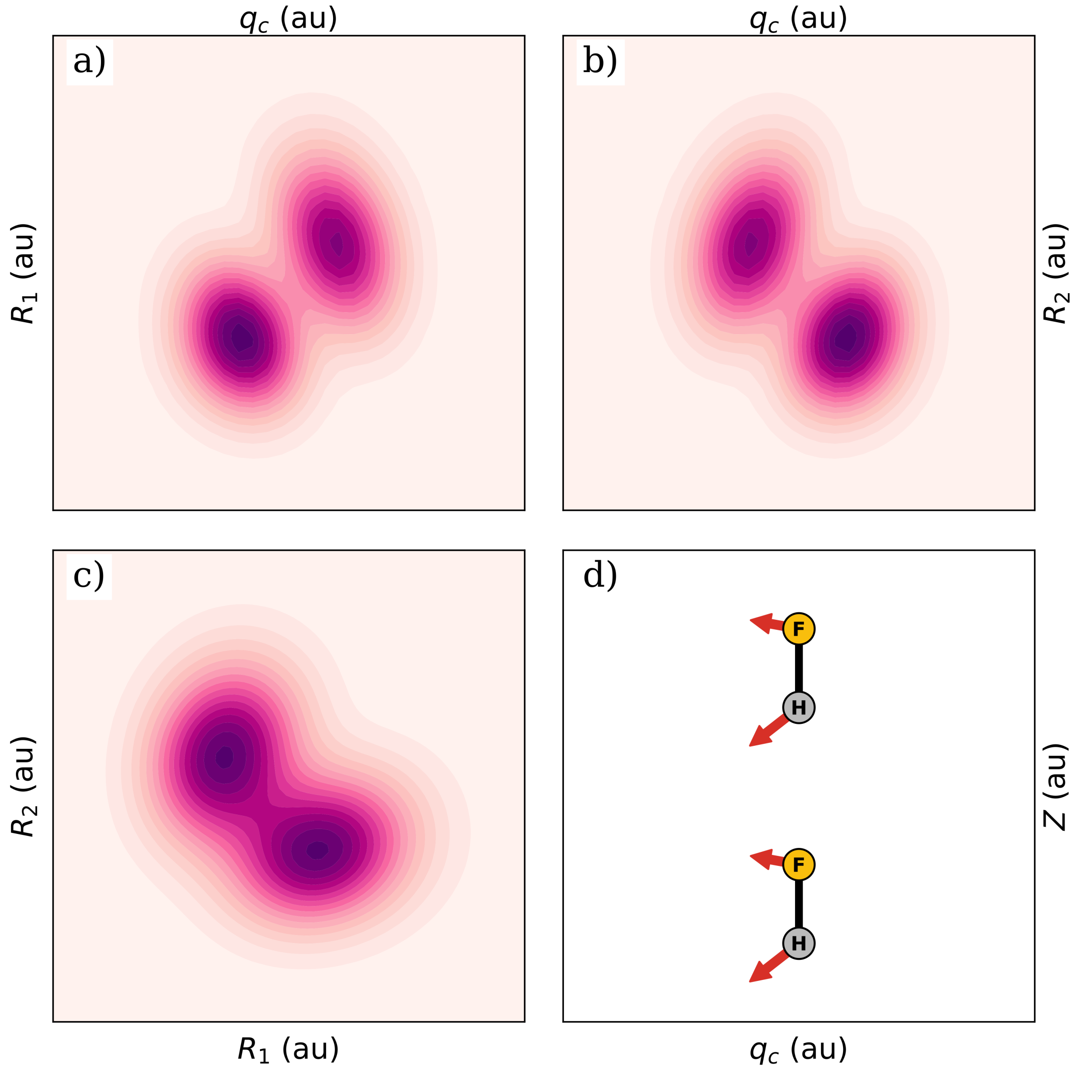

Figure S12: Third excited state eigenfunction for two parallel HF molecules described on a three-dimensional CBO-HF surface. For a), b), and c, the eigenfunction is integrated over one coordinate each. d) classic nuclear configuration and third mass-weighted normal mode of two parallel HF molecules. The molecules are separated by a distance of 800 Å. The cavity frequency ω_c is resonant with the corresponding fundamental transition (harmonic $4467\,\mathrm{cm}^{-1}$ and anharmonic $4281\,\mathrm{cm}^{-1}$). The coupling strength λ_c is set to 0.019 au.

S4 Beyond Diatomic Molecules: Vibro-Polaritonic Spectra of NH_3

The bare molecular vibronic IR spectrum and the vibro-polaritonic IR spectra of a single NH_3 molecule are shown in Fig. S13 for the three stretching modes. The cavity frequency ω_c is set to be resonant with the symmetric bending mode ν_1 ($1103\,\mathrm{cm}^{-1}$). The vibro-polaritonic IR spectra are calculated for the y polarization (Fig. S13 b) and d)) and the z polarization (Fig. S13 c) and d)) of the single cavity mode. Since the chosen cavity frequency w_c is off-resonant with the stretching modes, the cavity interaction leads only to modifications of the signals by changing the energetics and breaking the molecular symmetries. These effects are stronger for the y polarization because of the molecular orientation chosen.

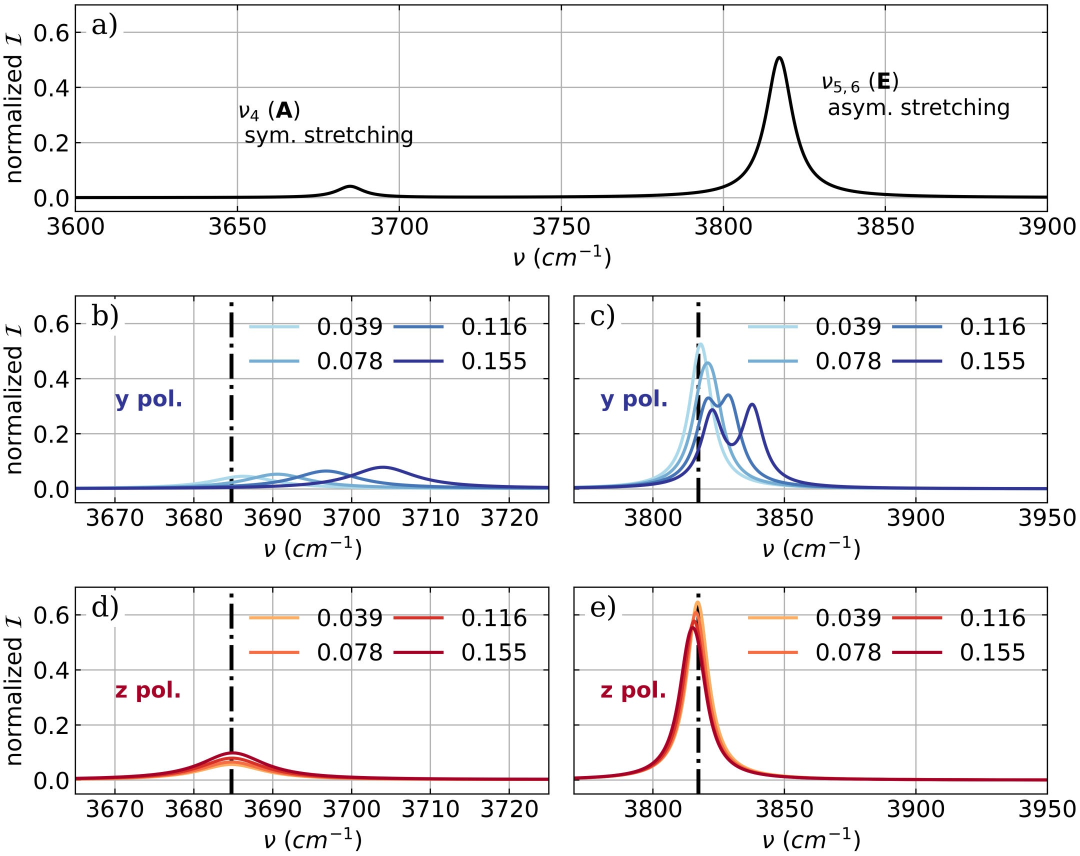

Figure S13: a) Vibronic IR spectra of a single NH_3 molecule calculated in the harmonic approximation. High energy part of the vibro-polaritonic IR spectra of a single NH_3 molecule zoomed into the symmetric stretching (b) and d)) and asymmetric stretching modes (c) and e)). The polarization axis of the cavity mode is the y axis for b) and c) and the z axis for d) and e). The cavity frequency ω_c is resonant with the symmetric bending mode ($1103\,\mathrm{cm}^{-1}$) and the cavity field strength λ_c is increased from $0.039\,\mathrm{au}$ to $0.155\,\mathrm{au}$.

References

(1) Pulay, P. Ab initio calculation of force constants and equilibrium geometries in polyatomic molecules. *Mol. Phys.* **1969**, *17*, 197–204.

(2) Pople, J. A.; Krishnan, R.; Schlegel, H. B.; Binkley, J. S. Derivative studies in hartree-fock and møller-plesset theories. *Int. J. Quantum Chem.* **2009**, *16*, 225–241.

(3) Jensen, F. *Introduction to computational chemistry*, 3rd ed.; John Wiley & Sons: Nashville, TN, 2017.

(4) Szabo, A.; Ostlund, N. S. *Modern Quantum Chemistry: Introduction to Advanced Electronic Structure Theory*, 1st ed.; Dover Publications, Inc.: Mineola, 1996.

(5) Bonini, J.; Flick, J. Ab Initio Linear-Response Approach to Vibro-Polaritons in the Cavity Born-Oppenheimer Approximation. *J. Chem. Theory Comput.* **2022**,

(6) Schnappinger, T.; Sidler, D.; Ruggenthaler, M.; Rubio, A.; Kowalewski, M. Cavity Born-Oppenheimer Hartree-Fock Ansatz: Light-Matter Properties of Strongly Coupled Molecular Ensembles. *J. Phys. Chem. Lett.* **2023**, *14*, 8024–8033.

(7) Fischer, E. W.; Saalfrank, P. Ground state properties and infrared spectra of anharmonic vibrational polaritons of small molecules in cavities. *J. Chem. Phys.* **2021**, *154*, 104311.
