# Supplementary material for: Correction to Ab Initio Vibro-Polaritonic Spectra in Strongly Coupled Cavity-Molecule Systems
Source: J Chem Theory Comput. 2024 Oct 31;20(21):9754–8. doi: 10.1021/acs.jctc.4c01374 (PMC11562371; doi:10.1021/acs.jctc.4c01374)
Supplement: Supplementary file 1 — ct4c01374_si_001.pdf [file ct4c01374_si_001.pdf]

Correction to "Supporting Information: Ab-Initio Vibro-Polaritonic Spectra in Strongly Coupled Cavity-Molecule Systems"

J. Chem. Theory Comput. 2023, 19, 24, 9278–9289; 10.1021/acs.jctc.3c01135)

Thomas Schnappinger* and Markus Kowalewski*

Department of Physics, Stockholm University, AlbaNova University Center, SE-106 91 Stockholm, Sweden

E-mail: thomas.schnappinger@fysik.su.se; markus.kowalewski@fysik.su.se

1 Corrected Figures in the Supporting Information

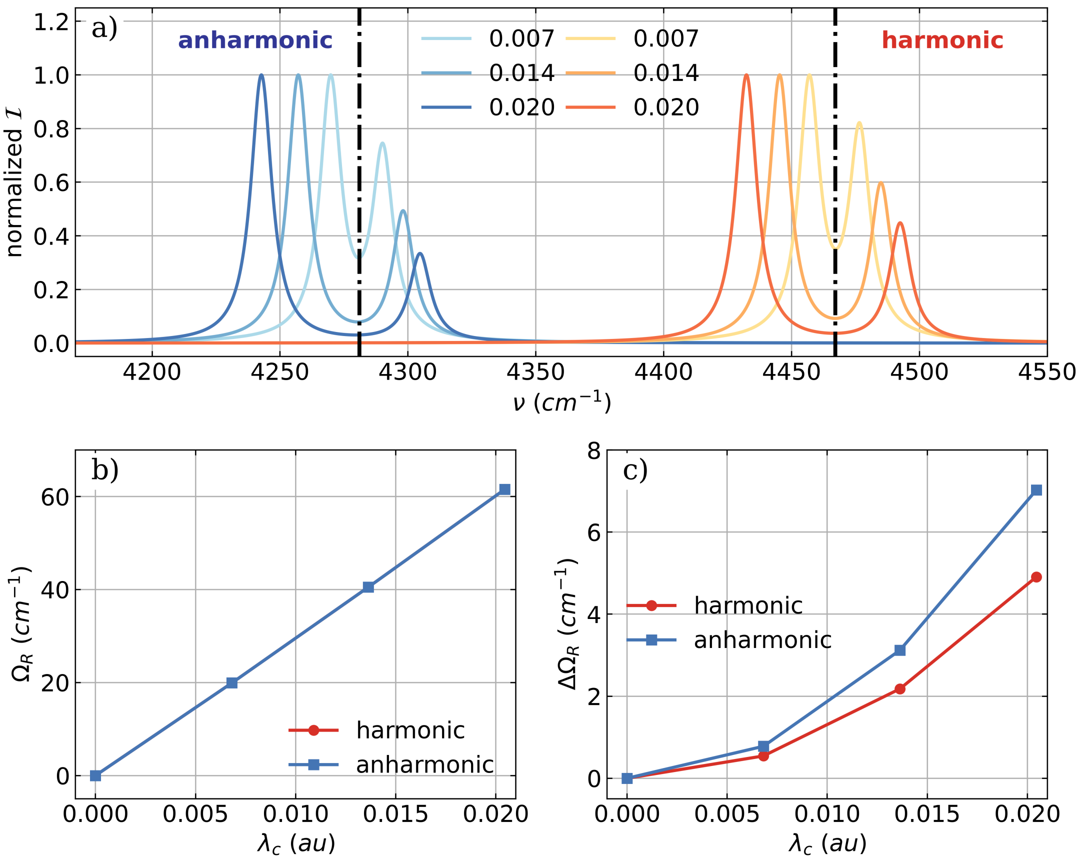

Figure S3: a) Vibro-polaritonic IR spectra of a two parallel HF molecule calculated in the harmonic approximation (reddish) and in the full anharmonic setup (bluish). Black dashed-dotted lines indicate the frequencies of the harmonic ($4467\,\mathrm{cm}^{-1}$) and anharmonic ($4281\,\mathrm{cm}^{-1}$) fundamental transitions. The cavity frequency ω_c is resonant with the corresponding fundamental transition in both cases, and the coupling strength λ_c is increased from $0.009\,\mathrm{au}$ to $0.039\,\mathrm{au}$ (from lightest to darkest color). b) Rabi splitting Ω_R as a function of λ_c. c) Asymmetry $\Delta\Omega_R = \omega_c - 0.5\left(\nu^{LP} + \nu^{UP}\right)$ of the Rabi splitting.

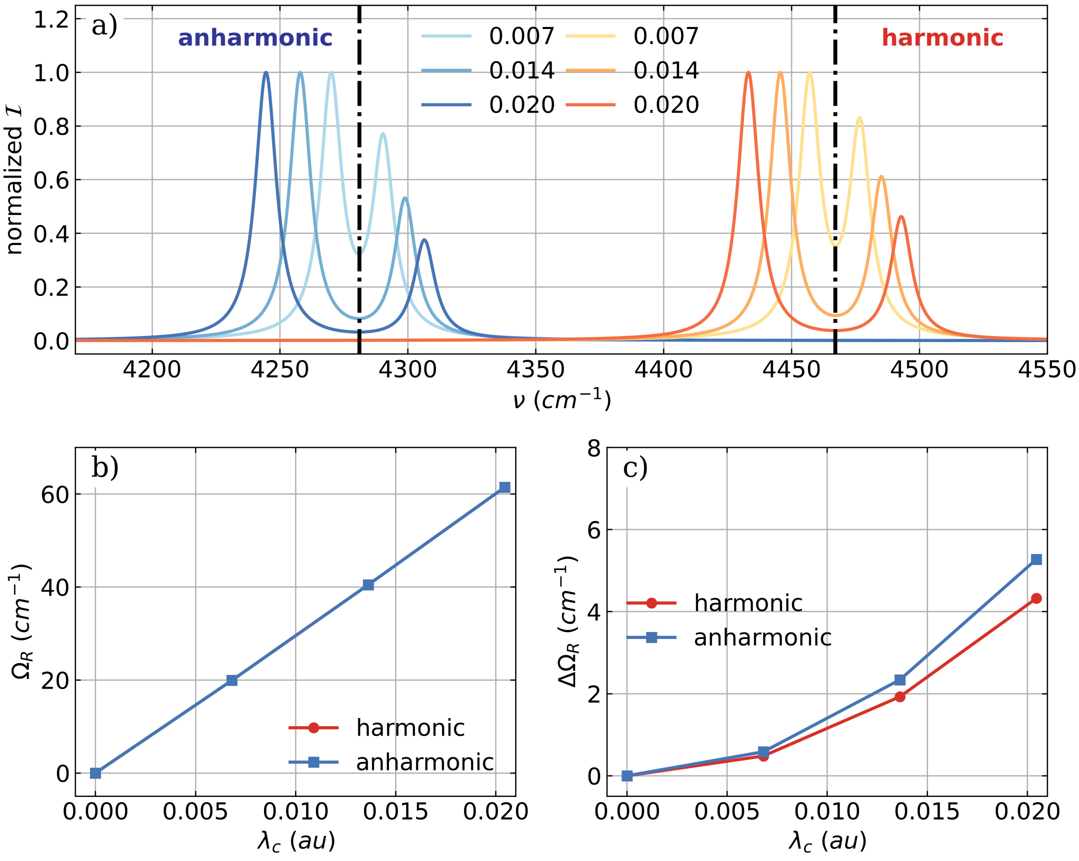

Figure S4: a) Vibro-polaritonic IR spectra of a two antiparallel HF molecules calculated in the harmonic approximation (reddish) and in the full anharmonic setup (bluish). Black dashed-dotted lines indicate the frequencies of the harmonic ($4467\,\mathrm{cm}^{-1}$) and anharmonic ($4281\,\mathrm{cm}^{-1}$) fundamental transitions. The cavity frequency ω_c is resonant with the corresponding fundamental transition in both cases, and the coupling strength λ_c is increased from $0.009\,\mathrm{au}$ to $0.039\,\mathrm{au}$ (from lightest to darkest color). b) Rabi splitting Ω_R as a function of λ_c. c) Asymmetry $\Delta\Omega_R = \omega_c - 0.5\left(\nu^{LP} + \nu^{UP}\right)$ of the Rabi splitting.

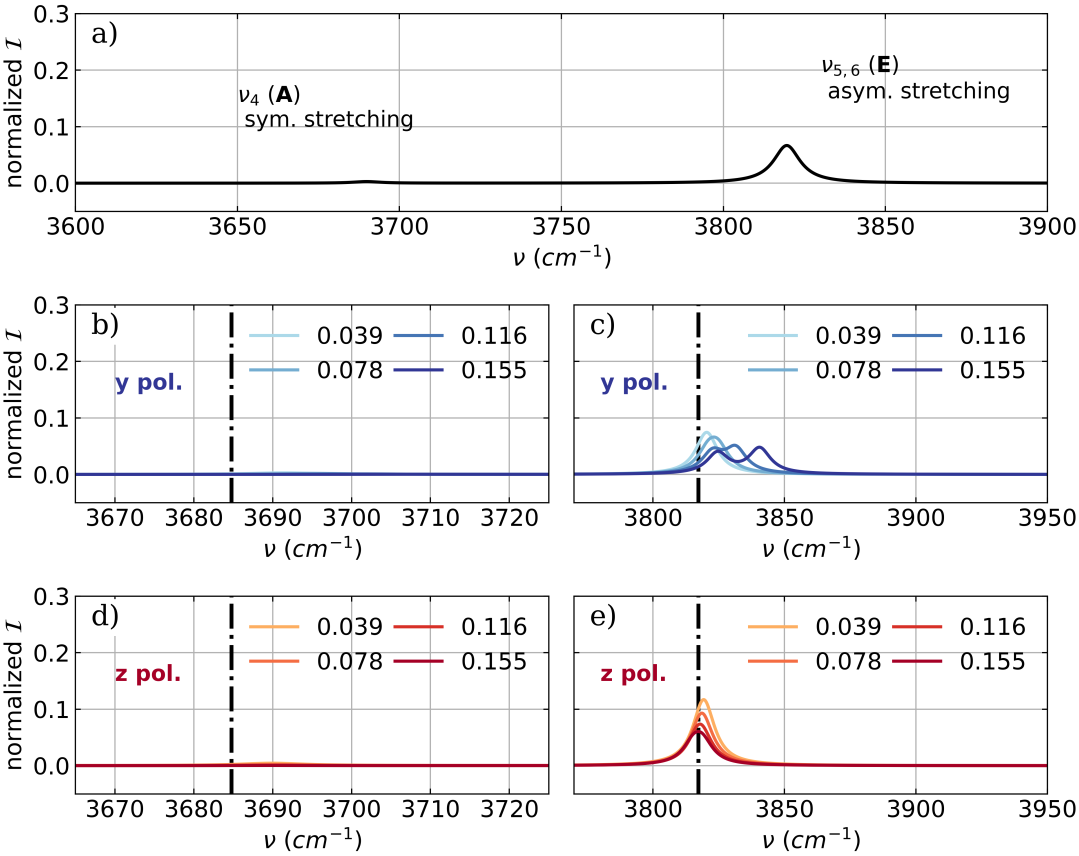

Figure S13: a) Vibrational IR spectra of a single NH_3 molecule calculated in the harmonic approximation. High energy part of the vibro-polaritonic IR spectra of a single NH_3 molecule zoomed into the symmetric stretching (b) and d)) and asymmetric stretching modes (c) and e)). The polarization axis of the cavity mode is the y axis for b) and c) and the z axis for d) and e). The cavity frequency ω_c is resonant with the symmetric bending mode ($1103\,\mathrm{cm}^{-1}$) and the cavity field strength λ_c is increased from $0.039\,\mathrm{au}$ to $0.155\,\mathrm{au}$.
